# SPSI: A Novel Composite Index for Estimating Panicle Number in Winter Wheat before Heading from UAV Multispectral Imagery

**DOI:** 10.34133/plantphenomics.0087

**Published:** 2023-09-06

**Authors:** Yapeng Wu, Wenhui Wang, Yangyang Gu, Hengbiao Zheng, Xia Yao, Yan Zhu, Weixing Cao, Tao Cheng

**Affiliations:** ^1^ National Engineering and Technology Center for Information Agriculture (NETCIA), MARA Key Laboratory for Crop System Analysis and Decision Making, MOE Engineering Research Center of Smart Agriculture, Jiangsu Key Laboratory for Information Agriculture, Nanjing Agricultural University, One Weigang, Nanjing, Jiangsu 210095, PR China.; ^2^ Langfang Normal University, 100 Aimin West Road, Langfang, Hebei 065000, PR China.

## Abstract

Rapid and accurate estimation of panicle number per unit ground area (PNPA) in winter wheat before heading is crucial to evaluate yield potential and regulate crop growth for increasing the final yield. The accuracies of existing methods were low for estimating PNPA with remotely sensed data acquired before heading since the spectral saturation and background effects were ignored. This study proposed a spectral-textural PNPA sensitive index (SPSI) from unmanned aerial vehicle (UAV) multispectral imagery for reducing the spectral saturation and improving PNPA estimation in winter wheat before heading. The effect of background materials on PNPA estimated by textural indices (TIs) was examined, and the composite index SPSI was constructed by integrating the optimal spectral index (SI) and TI. Subsequently, the performance of SPSI was evaluated in comparison with other indices (SI and TIs). The results demonstrated that green-pixel TIs yielded better performances than all-pixel TIs apart from TI_[HOM]_, TI_[ENT]_, and TI_[SEM]_ among all indices from 8 types of textural features. SPSI, which was calculated by the formula DATT_[850,730,675]_ + NDTI_COR[850,730]_, exhibited the highest overall accuracies for any date in any dataset in comparison with DATT_[850,730,675]_, TI_NDRE[MEA]_, and NDTI_COR[850,730]_. For the unified models assembling 2 experimental datasets, the *R*_V_^2^ values of SPSI increased by 0.11 to 0.23, and both RMSE and RRMSE decreased by 16.43% to 38.79% as compared to the suboptimal index on each date. These findings indicated that the SPSI is valuable in reducing the spectral saturation and has great potential to better estimate PNPA using high-resolution satellite imagery.

## Introduction

Wheat (*Triticum aestivum* L.) is one of the most widely cultivated cereal crops worldwide, and its yield is vital for global food security and sustainable development [1]. As one of the 3 components of yield, panicle number per unit ground area (PNPA) contributes the most to the final yield of wheat [2]. PNPA is a crucial yield phenotyping trait and has been widely concerned by crop growers and breeders. The acquisition of PNPA can help us better evaluate the planting density and improve the yield potential of wheat [3]. The traditional acquisition of PNPA by in situ manual counting is accurate but time-consuming and labor-intensive [4], which seriously limits its application in breeding, precision crop management, and yield estimation. With the development of remote sensing and image processing technologies, it is possible to obtain plot- and field-scale PNPA quickly and accurately in a nondestructive way [4,5]. Therefore, the rapid and accurate estimation of wheat PNPA from remote sensing imagery is important to coordinate yield components, increase yield, and accelerate breeding process.

At present, many studies have been reported on estimating the PNPA of wheat from remotely sensed data [5,6]. The majority of these studies focused on near-ground platforms, such as handheld cameras [7] and ground phenotyping platforms [8]. These platforms can be used to obtain accurate estimates of PNPA but cannot be operated over large areas due to low efficiency. Only a few studies estimated wheat PNPA with satellite imagery because of the low accuracy resulting from coarse resolutions [6]. In the recent decade, since unmanned aerial vehicles (UAV) have strong flexibility in obtaining high spatial-temporal resolution imagery, some researchers have been keen to estimate wheat PNPA from UAV imagery [9,10]. Nevertheless, they mainly used computer vision methods to count wheat panicles from RGB images acquired after heading [4,11]. These methods focusing on the post-heading stages (e.g., anthesis and filling) could contribute to reducing the workload of yield measurements and accelerating the breeding process for breeders [9,11]. Furthermore, the accuracy of panicle counting with RGB imagery is higher than that of panicle estimation with spectral imagery [8,12]. However, the panicle counting approaches based on RGB imagery with a higher spatial resolution (cm) cannot be extended to satellite imagery whose pixel size is currently far bigger than the size of a wheat panicle [13]. In contrast, it is more valuable to estimate wheat PNPA before heading for actual crop production from UAV multispectral sensors with lower cost than hyperspectral sensors and more bands than RGB cameras. This practice could help crop growers to manage and regulate field- and farm-scale PNPA in time for increasing the final yield of wheat, since the growth from the tillering stage to the booting stage determines the formation and number of wheat panicles [14]. Additionally, it is also expected to be applied to satellite platforms for guiding crop production in larger regions. Consequently, it becomes necessary to estimate wheat PNPA accurately using UAV multispectral imagery acquired before heading.

For decades, many researchers have favored optical remote sensing of crop traits that characterize crop growth status [15–17]. However, the spectral estimation of these parameters [e.g., aboveground biomass (AGB) and leaf area index] is confronted with the problem that the canopy reflectance tends to be saturated to various degrees in high vegetation densities [18]. Some spectral indices (SIs), such as the normalized difference vegetation index (NDVI) and the soil-adjusted vegetation index (SAVI), tended to be saturated after the AGB (range: 0 to 1.4 kg/m^2^) was greater than 0.4 kg/m^2^ when estimating wheat biomass [19]. This phenomenon was called the spectral saturation [20]. Since tiller number (TN) was related to AGB linearly and to PNPA nonlinearly at individual stages (Fig. S1), the spectral saturation phenomenon also exists in the estimation of wheat PNPA. To date, no research has paid specific attention to this phenomenon and established effective approaches to solve this problem. As a consequence, it is valuable to develop effective methods for reducing the spectral saturation in the estimation of wheat PNPA.

As an important image processing technology, textural analysis could measure the variability in pixel values between adjacent pixels in a defined analysis window [21]. Textural analysis with this property has been widely used for image classification [21,22]. Since the early 21st century, textural analysis has been successfully applied to estimate forest biomass [23]. In recent years, it has also been successfully used in estimating crop traits, such as AGB and plant potassium accumulation [24,25]. Although the combination of spectral and textural information has been widely used for improving the estimation accuracy of crop growth parameters, the respective contributions of these 2 types of information remain unclear [26,27]. Some researchers found that constructing a composite index through simple mathematical operation could not only combine the advantages of 2 types of information but also facilitate our understanding of their respective contributions [28,29]. Additionally, the composite indices fusing SIs and single-band textural features produced stronger resistance to saturation than SIs in estimating wheat AGB [29]. Since the textural indices (TIs) with more bands yielded better performances than the single-band textural features in estimating biomass [16], constructing a composite index by SI and TI integration has the potential to solve the spectral saturation problem for improving wheat PNPA estimation.

In addition, the canopy reflectance is seriously affected by significantly exposed background materials at the early stages of crop growth [15,30]. The contribution of textural features may also be affected by the exposed background materials, but current studies on textural analysis in crop monitoring ignored the background effect [26,31]. Although Guo et al. [32] have used the spectral and textural features derived from green pixels to identify the tasseling date of summer maize, it is still unknown whether and what differences exist between all-pixel textural features and green-pixel textural features. Hence, further research is needed to determine the necessity of background removal when estimating wheat PNPA using textural features from UAV multispectral imagery.

Therefore, we investigated the effect of background removal on TI performance and further determined the optimal SI and TI for constructing a new composite index sensitive to PNPA before heading in winter wheat. The specific objectives of this study were (a) to develop a composite index by integrating the optimal SI and TI for reducing the spectral saturation, (b) to evaluate the sensitivity of the relationship of the proposed composite index with PNPA to cultivation factors, and (c) to determine the optimal timing for estimating PNPA with the proposed composite index with the individual and pooled datasets.

## Materials and Methods

### Experimental design

Two field-trial experiments were conducted in the Comprehensive Demonstration Base for Agricultural Science and Technology located in Suining County, Jiangsu Province, China (35°56′N, 117°54′E) for winter wheat (*T. aestivum* L.) during the growing season in 2020 to 2021. The experimental site was characterized by a warm temperate continental monsoon climate. The accumulated precipitation, average temperature, and accumulated sunshine duration from 2020 October 20 to 2021 June 5 (the wheat growing season) were 271.5 mm, 10.5 °C, and 1103.3 h, respectively (Fig. S2). After harvesting the rice in this rice-winter wheat rotation system, the straw was crushed and returned to the field. The soil type was loam soil. Before sowing, the 0- to 20-cm soil layer exhibited a pH of 7.9 and contained 21.7 g/kg organic matter, 1.45 g/kg total nitrogen (N), 30.2 mg/kg available phosphorus (P), and 127.0 mg/kg available potassium (K).

The experiments involved 2 combinations of cultivars, N fertilizer rates, planting densities, and sowing dates with 3 replications (Table 1). The leaf types of wheat cultivars are erect (V1, V6, V7, and V9) and droop (V2, V3, V4, V5, and V8). Meanwhile, the above erect-leaf and droop-leaf cultivars are characterized by semi-winterness and springness, respectively. Experiment 1 (Exp. 1) used a split block design with cultivar as the main plot factor and N fertilizer rate as the subplot factor (Fig. S3A). Experiment 2 (Exp. 2) was arranged in a 3-factor strip-split block design with N fertilizer rate and sowing date as the main plot factors and planting density as the subplot factor (Fig. S3B). The number of plots in Exp. 1 and Exp. 2 was 48 and 81, respectively. For example, Fig. S4 exhibits the growth states of different wheat cultivars for N30 in Exp. 1 and different sowing dates for D15 and N30 in Exp. 2 in the field on 2021 March 2. The N fertilizers were applied in the form of urea by 50% as basal fertilization before sowing and 50% at the jointing stage. The P and K fertilizers were applied with 120 kg/ha P_2_O_5_ and 135 kg/ha K_2_O as basal fertilization before sowing. Other management practices were consistent with local recommendations for winter wheat production.

**Table 1.** Summary of designs for the 2 field plot experiments.

Exp. no.	Sowing date	Cultivar	Nitrogen rate (kg/ha)	Density (×10^4^ plants/ha)	Plot size (m)	Row spacing (cm)
Exp. 1 (VE)	2020 October 28 (S2)	Huaimai 33 (V1) Yangmai 23 (V2) Yangfumai 4 (V3) Ningmai 13 (V4) Yangmai 16 (V5) Jimai 22 (V6) Yannong 19 (V7) Zhenmai 12 (V8)	150 (N15) 300 (N30)	180 (D12)	5 × 6	35
Exp. 2(SE)	2020 October 20 (S1) 2020 October 30 (S2) 2020 November 10 (S3)	Xumai 33 (V9)	0 (N0) 240 (N24) 300 (N30)	225 (D15) 375 (D25) 525 (D35)	3 × 2	25

Note: VE represents the interaction of variety and nitrogen for Exp. 1. SE represents the interaction of sowing date, density, and nitrogen for Exp. 2.

### Data collection and preprocessing

#### Acquisition of UAV image and PNPA

Multispectral images over the experimental fields were obtained at all critical growth stages (regreening to maturity) of wheat using a 6-rotor UAV system (DJI M600 Pro, Shenzhen, China) equipped with an AIRPHEN camera (HI-PHEN, France) (Table S1). The AIRPHEN multispectral camera consisted of 6 global shutter sensors with filtering centers at 450, 530, 570, 675, 730, and 850 nm for a spectral resolution of 10 nm. The focal length lens of the camera was 8 mm. The field of view of the camera was 33.4° and 25.4° in the horizontal and vertical directions, respectively. The images with a size of 1280 × 960 pixels were continuously obtained at a frequency of 1 Hz and saved in TIFF format.

Our flight plans for 2 experiments were designed in the DJI GS Pro application software (https://www.dji.com/cn/ground-station-pro/). The UAV was set to automatic flying mode at nadir observation to acquire images with an across-track overlap of 95% and an along-track overlap of 90%. The flight altitude and the flight speed were 30 m (corresponding to a pixel size of 1.35 cm) and 3 m/s, respectively. All flights were carried out between 10:00 and 14:00 local time under clear-sky and stable light conditions. The influence of the solar zenith angle was not considered, due to the shorter flight time (about 7 min). During the filling stage, 4-row wheat plants within 1.4 m^2^ for Exp. 1 and 1 m^2^ for Exp. 2 were randomly selected in each plot to count the total panicle numbers, which were then converted to PNPA (× 10^4^/ha). Table S2 lists statistical information on the PNPA of winter wheat for 2 field plot experiments. The average PNPAs were significantly different (*P* < 0.05) across N rates or densities.

#### UAV image preprocessing

The UAV images were preprocessed within Agisoft PhotoScan Pro software (version 1.4.3, Agisoft LLC, St. Petersburg, Russia) to generate orthomosaics. The workflow of multispectral image preprocessing consisted of image alignment, geometric correction, camera optimization, construction of dense point clouds, mesh generation, radiometric calibration, and orthomosaic generation. Specifically, image alignment was done by feature point matching. A total of 29 circular panels (15 for Exp. 1 and 14 for Exp. 2) with a 30 cm diameter were adopted as ground control points (GCPs) for geometric correction (Fig. S3). Trimble GeoExplorer 6000 Series GeoXH (Trimble Navigation, Sunnyvale, CA, USA) was used to measure the position of GCPs within an error of 2.5 cm. Since the top of the wheat canopy was sharp, “Mild” depth filtering was chosen to build dense point clouds. A 3 m × 1 m rectangular gray panel was used to transform digital numbers into reflectance values for radiometric calibration [15,33]. The reference coefficients of 6 bands of multispectral imagery were 7.008% (450 nm), 7.291% (530 nm), 7.239% (570 nm), 7.442% (675 nm), 7.667% (730 nm), and 8.433% (850 nm), respectively.

### Derivation of SIs and TIs

#### Calculation of existing SIs

Several SIs, which have been commonly used to estimate crop biophysical and biochemical parameters, were selected from the literature to predict the PNPA of wheat (Table 2). Canopy reflectance is easily affected by the optical properties of background materials (soil and weeds in dry fields; wet soil, water, and duckweed in paddy fields), especially before canopy closure, which often resulted in the low estimation accuracy of crop biophysical and biochemical parameters [15,34]. Vegetation index thresholding is a direct and effective method to remove the background pixels from UAV imagery [33]. The reflectance images after removing the background pixels could be used not only to extract green-pixel reflectance but also to generate green-pixel textural features. Additionally, the use of green-pixel reflectance images improved the efficiency of deriving texture as compared to the use of all-pixel ones (Table S3). Therefore, the vegetation index thresholding method was adopted to remove the background and extract green pixels (wheat pixels alone). Additionally, the visible atmospherically resistant index (VARI) derived from the visible region has proved to be sensitive to vegetation fraction [35]. According to the reflectance characteristics of soil and weeds (chickweed mainly) in the experimental field, the green band (530 nm) with a threshold of 0.063 and the VARI with a threshold of 0.08 were used to extract wheat pixels from UAV imagery on 2021 February 23 (Fig. S5). For the images from other dates, the thresholds for extracting wheat pixels were floating (VE: *R*_530_ = 0.052 to 0.078, VARI = −0.21 to 0.08; SE: *R*_530_ = 0.052 to 0.088, VARI = −0.02 to 0.10). After a region of interest (ROI) was established manually for each plot, the average spectral reflectance over wheat pixels within each ROI was extracted for calculating all SIs. The whole process was carried out by using the ENVI software (version 5.3, Exelis Visual Information Solutions, Boulder, CO, USA).

**Table 2.** Selected spectral indices used in this study.

Index	Full name	Formulation	Reference
NDVI	Normalized difference vegetation index	(*R*_850_ − *R*_675_)/(*R*_850_ + *R*_675_)	Rouse et al. [20]
NDRE	Normalized difference red edge	(*R*_850_ − *R*_730_)/(*R*_850_ + *R*_730_)	Fitzgerald et al. [66]
EVI2	Two-band enhanced vegetation index	2.5 × (*R*_850_ − *R*_675_)/(1 + *R*_850_ + 2.4 × *R*_675_)	Jiang et al. [67]
CI_green_	Green chlorophyll index	*R*_850_/*R*_530_ − 1	Gitelson et al. [68]
CI_red-edge_	Red-edge chlorophyll index	*R*_850_/*R*_730_ − 1	Gitelson et al. [68]
NEI	Nitrogen efficiency index	[(1.8 + *R*_675_/*R*_850_) × *R*_730_ + *R*_675_]/(*R*_850_ + *R*_675_)	Zhang et al. [45]
OSAVI	Optimized soil-adjusted vegetation index	(*R*_850_ − *R*_675_)/(*R*_850_ + *R*_675_ + 0.16)	Rondeaux et al. [34]
DATT_[850,730,675]_	(*R*_850_ − *R*_730_)/(*R*_850_ − *R*_675_)	(*R*_850_ − *R*_730_)/(*R*_850_ − *R*_675_)	Datt [69]
TBVI_[850,730,450]_	Three-band vegetation index	(*R*_850_ − *R*_730_ + 2 × *R*_450_)/(*R*_850_ + *R*_730_ – 2 × *R*_450_)	Wang et al. [49]

Note: The formulations of SIs were derived from references, but the use of wavebands was based on the AIRPHEN multispectral camera. *R*_850_, *R*_675_, *R*_730_, and *R*_530_ represent the reflectance values at 850, 675, 730, and 530 nm, respectively.

#### Textural analysis and calculation of TIs

Approaches for deriving texture can be mainly divided into 4 categories: statistical methods, structural methods, model-based methods, and transform-based methods [36]. Gray-level co-occurrence matrix (GLCM), a typical representative of the statistical methods, is the most popular and widely used method [16,25]. The GLCM could reveal certain properties about the spatial distribution of the pixels in gray levels presented at a certain moving distance and a particular moving direction [21]. The GLCM was adopted to derive texture, and the operations were conducted in the ENVI environment.

Considering the 1.35-cm pixel size of the UAV images relative to the leaf width of 0.4 to 1.9 cm before heading, the GLCMs were operated with a 1-pixel moving distance and the smallest moving window (3 × 3 pixels). To reduce the computational effort and avoid creating sparse GLCMs [37], all GLCMs were constructed using grayscale quantization levels of 64. When calculating the GLCM, there are usually 4 moving directions to choose from 0°, 45°, 90°, and 135° [21]. Several studies have shown that the optimal angle varies with crop type and row orientations for textural features generated by the GLCM, but there were only slight differences among the 4 moving directions [26,32,38]. The angle 45° was chosen as the moving direction as used in [38] with the south–north row orientation of winter wheat. Eight of the most commonly used textural features [21,39] were computed, including mean (MEA), variance (VAR), homogeneity (HOM), contrast (CON), dissimilarity (DIS), entropy (ENT), angular second-moment (SEM), and correlation (COR) (Table S4). These textural features were generated from the reflectance orthomosaics of all pixels (wheat and background pixels) [33] and green pixels (wheat pixels alone), respectively. The use of green-pixel reflectance orthomosaics improved the efficiency of GLCM computation as compared to the use of all-pixel ones (Table S3). The textural analysis produced 16 sets of textural images (Fig. S6). The same ROIs from the reflectance images were applied to these textural images. The average values within each ROI were extracted as the textural features of each plot.

As the textural features are single-band outputs from the texture analysis, they can be used to develop TIs as done for SI calculation [40]. Although the form of TI would be diversified with the configuration of various bands and textural features, TIs were usually constructed with reference to the classical forms of the reported SIs [16,25]. Considering the popularity of all SI forms in Table 2, the TIs for 2-pixel compositions (all pixels or green pixels) were calculated based on the forms of the 4 SIs best correlated to PNPA from Table 2. Then, these derived TIs were used for determining the suitable textural variable.

#### Construction of SPSI for wheat PNPA estimation

While monitoring crop biomass at high levels, the canopy reflectance tended to be saturated [18]. Similarly, the spectral saturation phenomenon also exists in PNPA estimation due to the strong relationships of TN with AGB (linear) and PNPA (logarithmic) during individual stages (Fig. S1). Considering that texture could reflect the characteristics of vegetation structure [39], a TI was developed to reduce the spectral saturation in PNPA (structural parameter) estimation. As one of the 8 commonly used texture features, COR reflects the linear dependency of pixel values among neighboring pixels for the grayscale image [21] so that it could identify similar crop structures. Additionally, COR was closely related to the AGB and yield of winter wheat [31,41]. The traditional normalized difference form for SI construction has the advantage of eliminating most of the noise caused by instrument calibration, terrain, and cloud shadow [20,42]. Therefore, we proposed a normalized difference TI based on COR (NDTI_COR_) to reduce the spectral saturation in PNPA estimation as described by the following equation:

$${\text{NDTI}}_{\text{COR}\left[\lambda 1,\lambda 2\right]}=\frac{T_{\lambda 1}-T_{\lambda 2}}{T_{\lambda 1}+T_{\lambda 2}}$$ (1)

where *λ*1 and *λ*2 both represent the wavelengths of random bands in the UAV multispectral imagery. *T*_*λ*1_ and *T*_*λ*2_ are the COR values of band 1 (*λ*1) and band 2 (*λ*2), respectively.

Considering the difference between spectral and textural information [21,43], constructing the composite index by the additive operation is the simplest way to combine the advantages of these 2 types of information as compared to the multiplicative operation in relevant research [28]. In view of the construction of LAI-insensitive chlorophyll index (LICI) as the difference of 2 contrasting indices [44], the addition of 2 indices with consistently positive or negative correlations with PNPA could enhance the relationship between the composite index and PNPA. As a consequence, the spectral-textural PNPA sensitive index (SPSI) was developed to reduce the spectral saturation and improve the accuracy of PNPA estimation across the early growth stages as described below:

$$\text{SPSI}=SI_{\mathrm{OPT}}+{\text{NDTI}}_{\mathrm{COR}\left[\mathrm{OPT}\right]}$$ (2)

where SI_OPT_ and NDTI_COR[OPT]_ are the optimal SI and NDTI_COR_ sensitive to wheat PNPA estimation, respectively. Among the random combinations of spectral bands for constructing NDTI_COR_, the 2 ones with the strongest positive and negative correlations with PNPA would be determined, respectively. The positive or negative sign of the correlation between NDTI_COR[OPT]_ and PNPA was consistent with that between SI_OPT_ and PNPA.

### Statistical modeling and accuracy evaluation

Simple linear regression (SLR) was used to construct empirical relationships between remote sensing variables and PNPA. To develop robust models applicable to various cultivation conditions [17,45], the SE and VE datasets with different treatments were used collectively as the pooled dataset. Meanwhile, they were also used individually for evaluating the performance of SPSI under specific conditions. For data partitioning, two-thirds of each dataset (SE dataset or VE dataset) were randomly selected for modeling and the remaining for validation. The model accuracy was evaluated by the commonly used coefficient of determination (*R*_V_^2^), root mean square error (RMSE), and relative RMSE (RRMSE) calculated as below:

$$R_{V}^{2}=1-\frac{\sum_{i}{\left(y_{i}-y_{i}^{\prime }\right)}^{2}}{\sum_{i}{\left(y_{i}-\bar{y}\right)}^{2}}$$ (7)

$$\text{RMSE}=\sqrt{\frac{\sum_{i}{\left(y_{i}-y_{i}^{\prime }\right)}^{2}}{n}}$$ (8)

$$\text{RRMSE}=\frac{\text{RMSE}}{\bar{y}}\times 100\%$$ (9)

where *y_i_* and $y_{i}^{\prime }$ are the measured and predicted PNPA for sample *i*, respectively. $\bar{y}$ is the arithmetic mean of the measured PNPA over all samples, and *n* is the number of samples. Specifically, the $R_{V}^{2}$ used here is different from the squared correlation coefficient and a better measure of data fitting to the 1:1 line. The $R_{V}^{2}$ values may be negative, indicating that the estimated PNPA is worse than simply using the mean of the measured PNPA [46]. Following Li et al. [43], the negative $R_{V}^{2}$ values were set as zero to avoid confusion caused by severe overestimation or underestimation.

## Results

### Relationships of PNPA with SIs or TIs

**Fig. 1.**
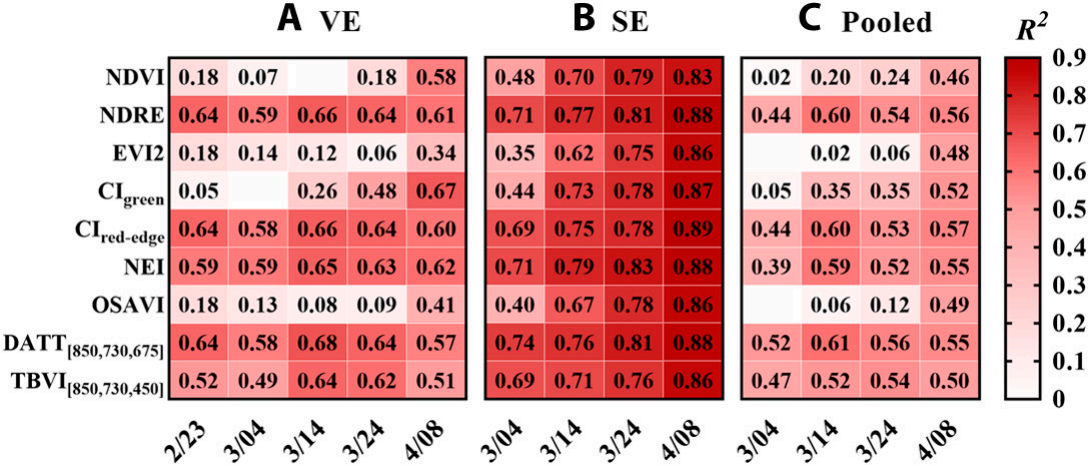
(A to C) Coefficient of determination (*R*^2^) values for linear relationships of PNPA with SIs over individual dates before heading for different datasets. The values of the valueless regions were lower than 0.00.

Figure 1 displays an *R*^2^ heatmap of the PNPA–SI relationships for different dates before heading in individual (VE and SE datasets) and pooled datasets. For any of the datasets, 4 better SIs of the 9 conventional SIs were most closely related to PNPA on all dates, namely, NDRE, CI_red-edge_, NEI (nitrogen efficiency index), and DATT_[850,730,675]_. Furthermore, DATT_[850,730,675]_ yielded the highest *R*^2^ values for the pooled dataset. The relationships between the 4 better SIs and PNPA varied with date for the individual datasets, and the *R*^2^ values increased and then decreased on the whole (Fig. S7). Specifically, the *R*^2^ values peaked on March 14 (the initial-jointing stage with reference to semi-winter wheat) for the VE dataset (Fig. S7A) and April 8 (the initial-booting stage with reference to wheat planted in S2) for the SE dataset (Fig. S7B). Moreover, there were only marginal differences in *R*^2^ from April 8 to May 9 (the booting stage to the initial-filling stage with reference to semi-winter wheat). The TIs based on the 4 better SI forms for 2-pixel compositions (all pixels and green pixels) exhibited significantly different performances in PNPA estimation for the March 14 portion of VE and SE datasets (Fig. S8). While TI_[HOM]_, TI_[ENT]_, and TI_[SEM]_ showed higher *R*^2^ values of all-pixel indices than those of green-pixel indices, the TIs based on the remaining textural features exhibited the opposite except for TI_DATT[850,730,675]_. In general, TI_NDRE[MEA]_ derived from green pixels gave the highest *R*^2^ among various TIs. Consequently, green pixels were selected for deriving textural features in subsequent analyses.

**Fig. 2.**
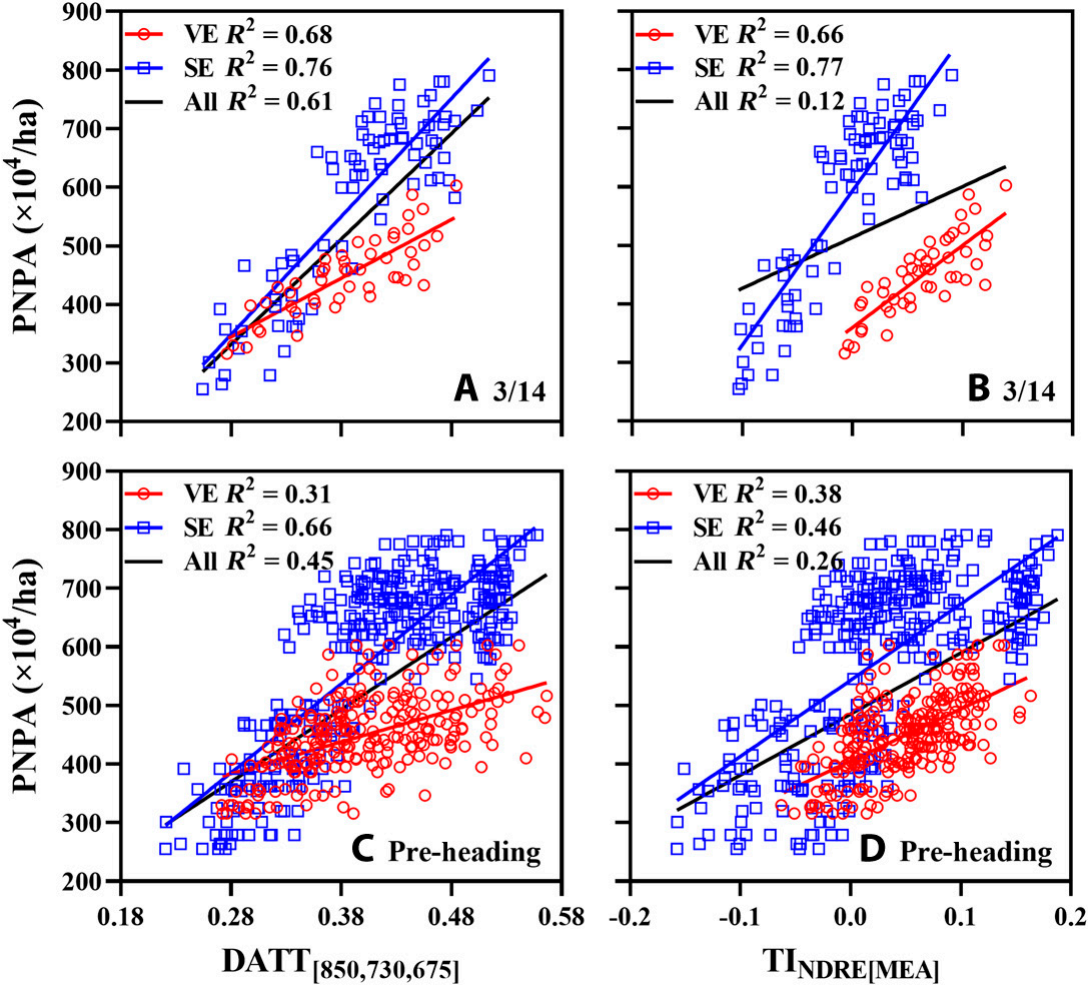
The relationships of PNPA with DATT_[850,730,675]_ (A and C) and TI_NDRE[MEA]_ (B and D) in winter wheat on different dates. Black lines represent the fitted lines of all data points.

Figure 2 shows the relationships of PNPA with DATT_[850,730,675]_ and TI_NDRE[MEA]_ for the March 14 and pre-heading portions of the individual and pooled datasets. Regardless of March 14 and the pre-heading stages, the obvious stratification phenomenon existed among the individual datasets of each group when applied to the pooled dataset in terms of DATT_[850,730,675]_, especially for the high-value range (Fig. 2A and C). In addition, the *R*^2^ values of TI_NDRE[MEA]_ (*R*^2^ = 0.66 for VE and 0.77 for SE; Fig. 2B) were similar to those of DATT_[850,730,675]_ (*R*^2^ = 0.68 for VE and 0.76 for SE; Fig. 2A) when estimating the PNPA from the March 14 portion of the individual datasets. However, TI_NDRE[MEA]_ showed more obvious stratification (*R*^2^ < 0.26; Fig. 2B and D) than DATT_[850,730,675]_ (*R*^2^ > 0.45; Fig. 2A and C) when applied to the March 14 or pre-heading portion of the pooled dataset.

### Determination of SPSI

**Fig. 3.**
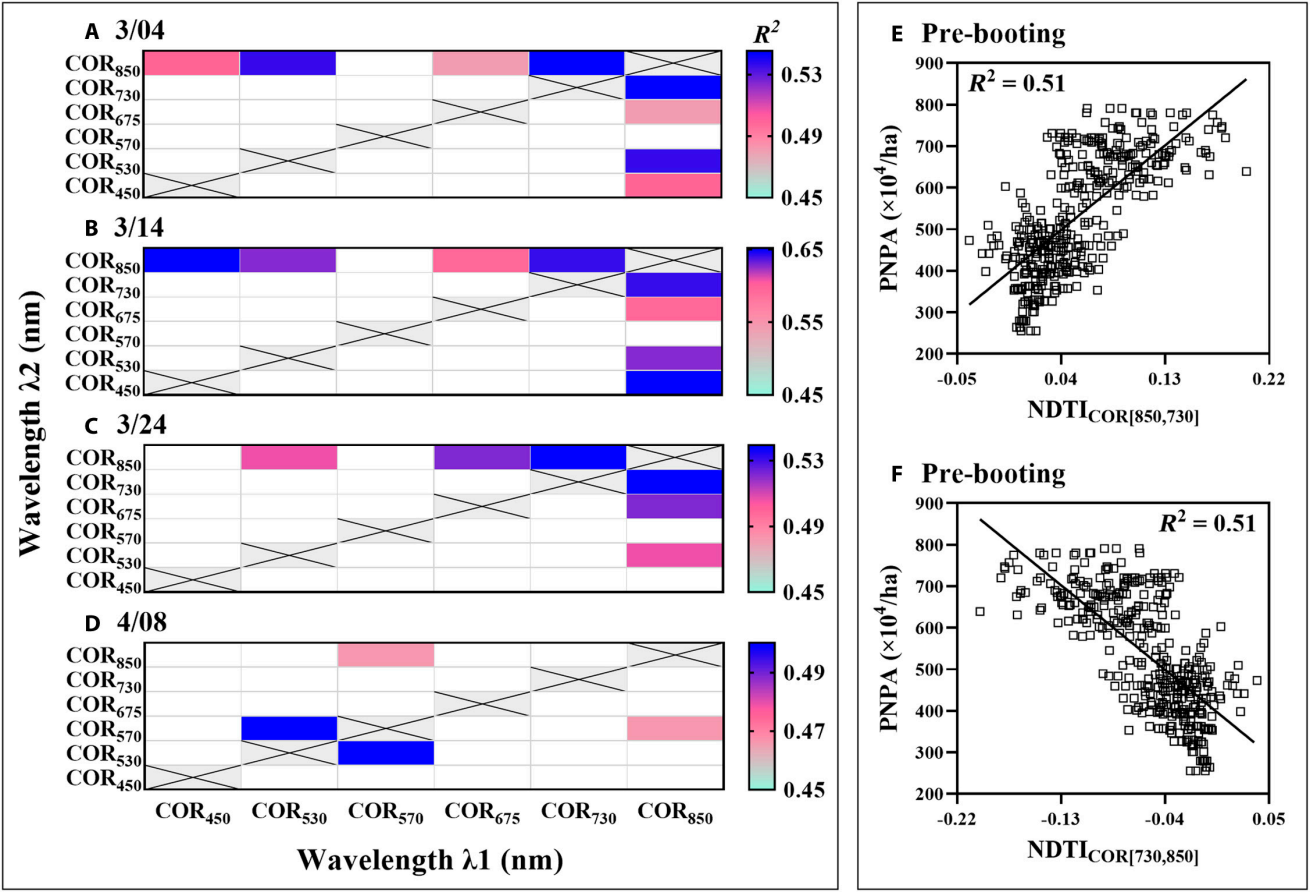
*R*^2^ for linear relationships between PNPA and NDTI_COR_ of all possible band combinations (A to D) and the relationships of PNPA with NDTI_COR[850,730]_ (E) and NDTI_COR[730,850]_ (F). The gray reticulated area represents invalid combinations, and the white area represents the band combinations with *R*^2^ lower than 0.45.

The combinations of common sensitive bands were determined from the pooled data for March 4, March 14, March 24, and April 8, respectively (Fig. 3A to D). For the lower right portion of each subfigure, the sensitive bands for λ1 appeared consistently at 850 nm across the first 3 dates. In addition, the sensitive bands for λ2 were inconsistent across the 4 dates but overlapped at 730 nm across the first 3 dates. By contrast, the upper left portion of each subfigure showed consistently sensitive bands at opposite band positions. Overall, the band combinations sensitive to PNPA were (a) [λ1: 850 nm, λ2: 730 nm] and (b) [λ1: 730 nm, λ2: 850 nm] for the pooled data from any of the first 3 dates. Besides, the April 8 portion of the pooled dataset produced 2 special combinations composed of 530 and 570 nm. In sum, the 2 COR-based NDTIs sensitive to PNPA before booting were denoted as NDTI_COR[850,730]_ and NDTI_COR[730,850]_, respectively. Moreover, they still had the strongest correlations with PNPA among the NDTIs from all possible band combinations for the pre-booting portion of the pooled dataset (Fig. S9). NDTI_COR[850,730]_ and NDTI_COR[730,850]_ exhibited opposite correlations with PNPA (Fig. 3E and F). The former was positively correlated with PNPA (Fig. 3E), which was similar to the relationship between DATT_[850,730,675]_ and PNPA (Fig. 2A and C). Consequently, the optimal NDTI_COR_ sensitive to PNPA was determined as NDTI_COR[850,730]_ (the same as TI_NDRE[COR]_). As a result, the pre-booting portion of the pooled dataset was selected for constructing SPSI in subsequent analyses.

**Fig. 4.**
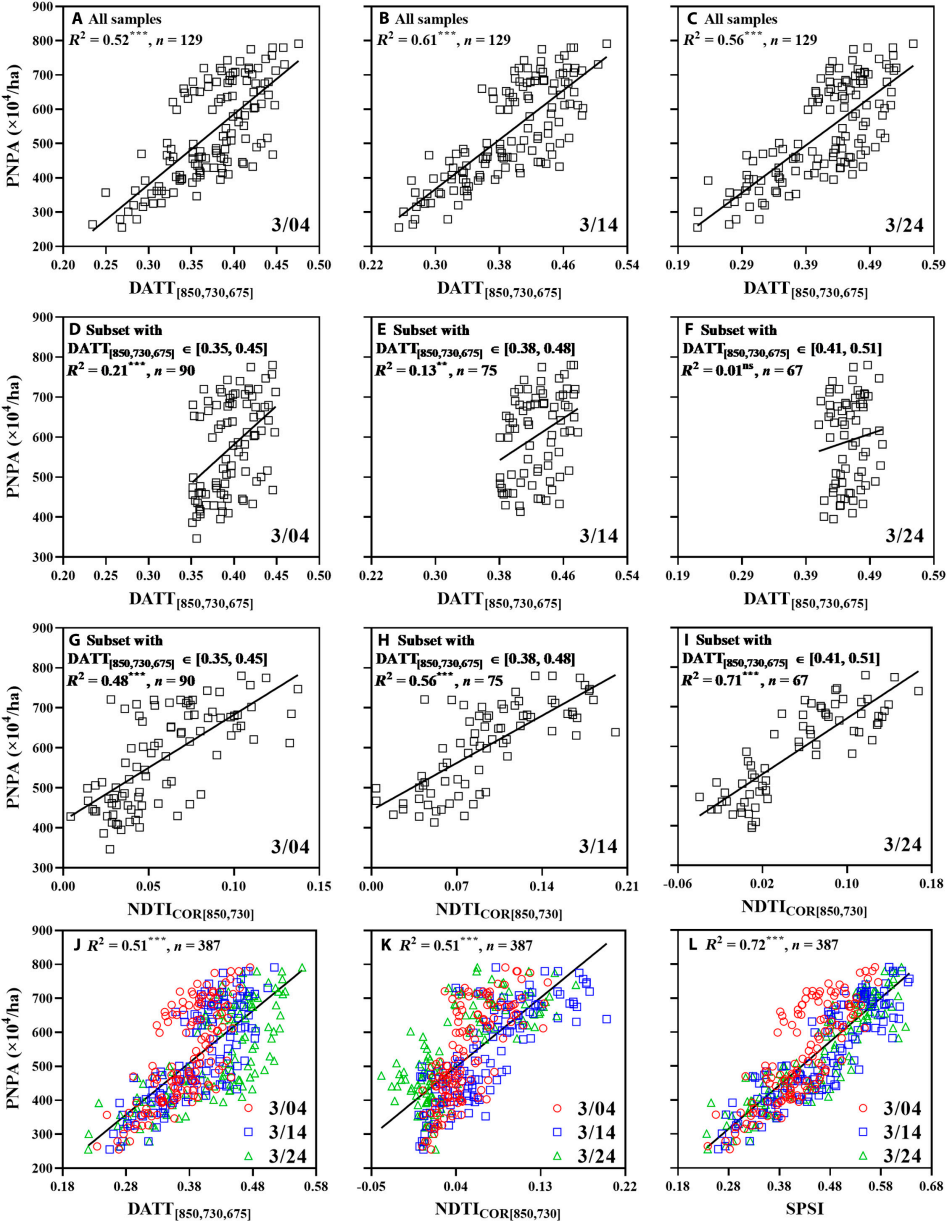
The relationships of PNPA with DATT_[850,730,675]_, NDTI_COR[850,730]_, and SPSI in winter wheat over individual dates (A to I) or across pre-booting stages (J to L). (A) to (C) are for all samples, and (D) to (F) are for subsets with DATT_[850,730,675]_ in the range of 0.35 to 0.45 (D), 0.38 to 0.48 (E), and 0.41 to 0.51 (F), respectively. (G) to (I) are for the subsets where the PNPA samples (plot numbers) are the same as those in (D) to (F), respectively. DATT_[850,730,675]_ and NDTI_COR[850,730]_ were used to derive SPSI. Significance level: no significance, ns; *P < 0.05; **P < 0.01; ***P < 0.001.

Furthermore, the ability of NDTI_COR[850,730]_ to reduce the spectral saturation when estimating PNPA could be observed in Fig. 4A to I. Specifically, DATT_[850,730,675]_ was strongly correlated with PNPA across all samples from any of the 3 dates (*R*^2^ = 0.517 to 0.615) (Fig. 4A to C). Nevertheless, the PNPA varied in a wide range [RV (range of variation) ≈ 400 to 800 ×10^4^/ha] and was weakly related to DATT_[850,730,675]_ for 3 subsets in the high-value region of DATT_[850,730,675]_, as a result of spectral uniformity with different PNPAs (Fig. 4D to F). When estimating PNPA in the above 3 subsets with NDTI_COR[850,730]_, stronger correlations with PNPA (*R*^2^ = 0.48 to 0.71) were produced than those with DATT_[850,730,675]_ (*R*^2^ < 0.21) (Fig. 4G to I). The relationships of PNPA with DATT_[850,730,675]_ and NDTI_COR[850,730]_ were further compared to construct SPSI as a composite index with the pre-booting portion of the pooled dataset (Fig. 4J to L). Both DATT_[850,730,675]_ and NDTI_COR[850,730]_ were positively correlated with PNPA. The addition of these 2 indices (SPSI, *R*^2^ = 0.72) was more closely related to PNPA than either DATT_[850,730,675]_ (*R*^2^ = 0.51) or NDTI_COR[850,730]_ (*R*^2^ = 0.51) alone.

### Sensitivity of SPSI to cultivation factors

**Fig. 5.**
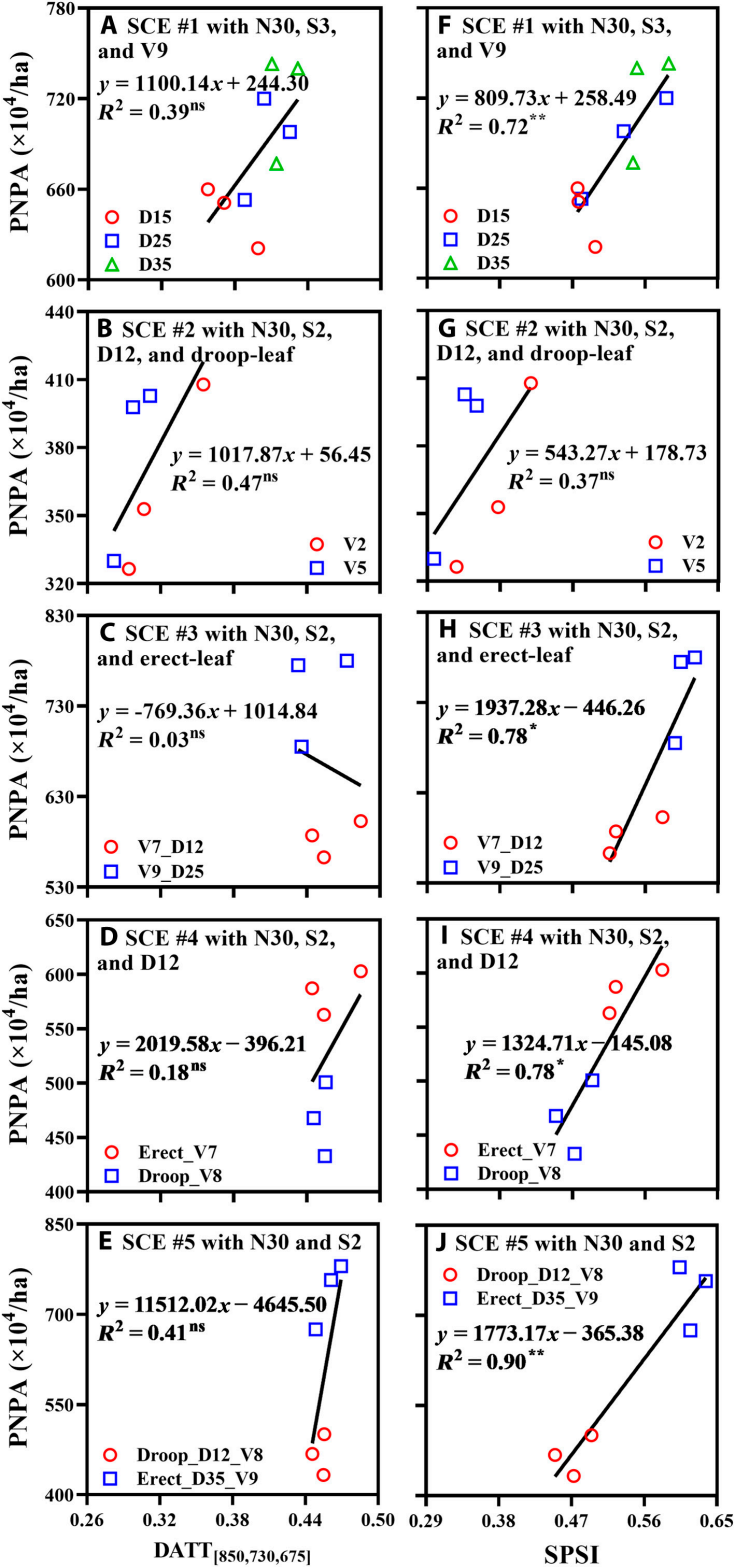
The relationships of PNPA with DATT_[850,730,675]_ (A to E) and SPSI (F to J) in winter wheat with 5 scenarios on 2021 March 14. (A and F) SCE #1 represents scenario #1 where the N level and sowing date are consistent but the densities are different for V9. (B and G) SCE #2 represents scenario #2 where the N level, sowing date, density, and leaf type are consistent but the cultivars are different. (C and H) SCE #3 represents scenario #3 where the N level, sowing date, and leaf type are consistent but the densities and cultivars are different. (D and I) SCE #4 represents scenario #4 where the N level, sowing date, and density are consistent but the leaf types and cultivars are different. (E and J) SCE #5 represents scenario #5 where the N level and sowing date are consistent but the densities, leaf types, and cultivars are different. Significance level: no significance, ns; *P < 0.05; **P < 0.01.

Figure 5 displays the sensitivities of SPSI to PNPA under 5 spectral uniformity scenarios (SCE #1 to SCE #5) for 5 combinations of cultivation factors. The PNPA values differed remarkably with nearly constant DATT_[850,730,675]_ (Fig. 5A to E). The fitting lines of data points exhibited no significance for SCE #1 to SCE #5 (Fig. 5A to E). By contrast, the relationships of SPSI with PNPA for these scenarios were much stronger (*R*^2^ > 0.72, *P* < 0.05) except for SCE #2 (Fig. 5F to J).

**Fig. 6.**
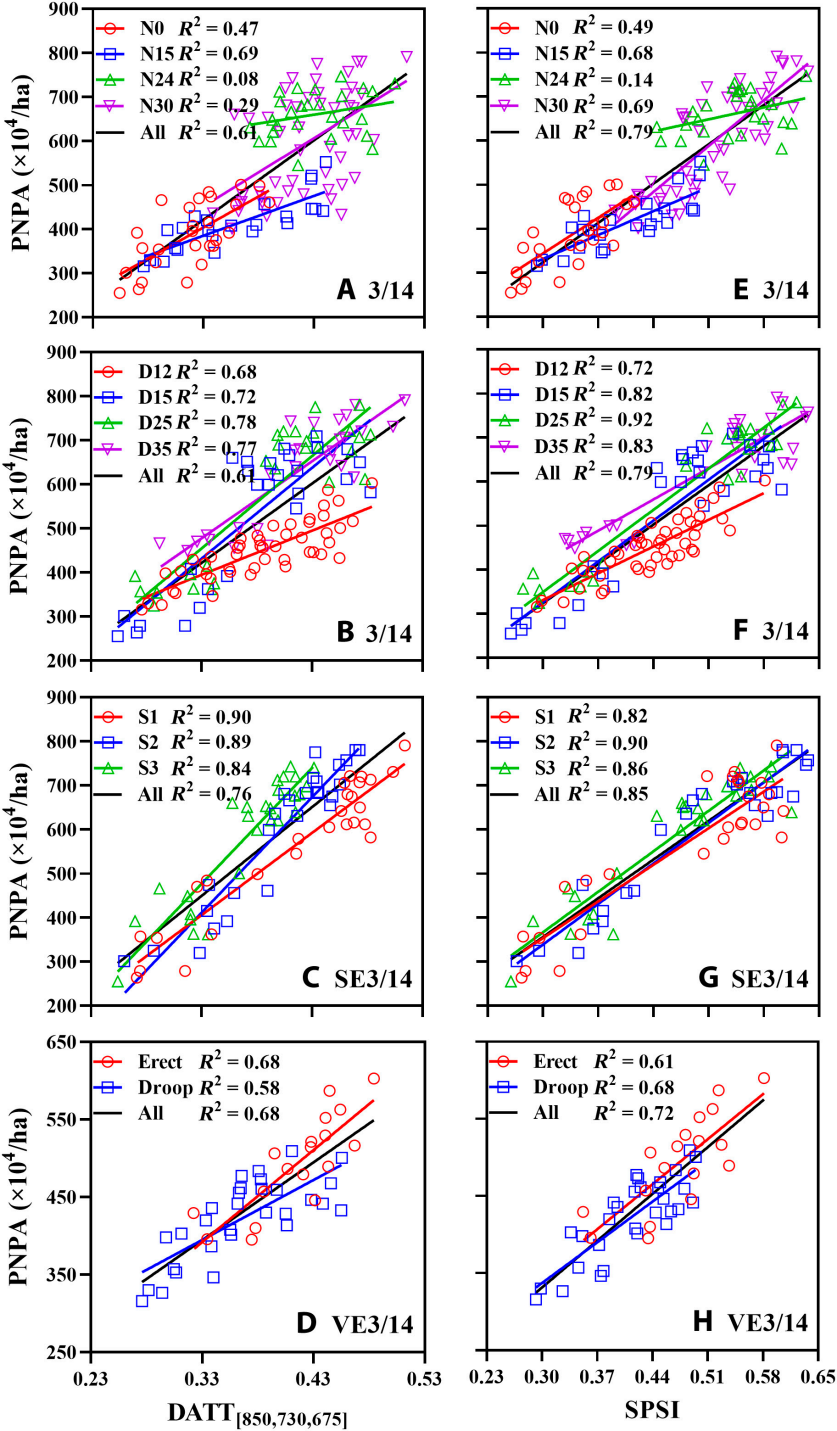
The relationships of PNPA with DATT_[850,730,675]_ (A to D) and SPSI (E to H) under different experimental conditions. (A) to (H) show analyses of separate datasets for nitrogen levels (A and E), planting densities (B and F), sowing dates (C and G), and leaf types (D and H). Black lines represent the fitted lines of all data points.

The regressions in Fig. 6 demonstrated that the relationships of PNPA with SPSI for the pooled data categorized by cultivation factors were stronger than those with DATT_[850,730,675]_, which suggests the lower sensitivities of SPSI to cultivation factors. However, the performances of SPSI varied across cultivation factors at different levels. Compared with DATT_[850,730,675]_ (*R*^2^ = 0.90 for S1 and 0.68 for Erect), SPSI exhibited worse performances for S1 (*R*^2^ = 0.82) and Erect (*R*^2^ = 0.61) but comparable (N0, N15, S2, and S3) or even better performances at other levels. Among them, the *R*^2^ value of SPSI increased by 0.40 as compared to DATT_[850,730,675]_ for N30. Generally, the *R*^2^ values between SPSI and PNPA were greater than 0.61 except for N0 and N24 (Fig. 6E to H).

### Modeling and validation

The SLR models and *R*^2^ between 4 indices and PNPA are displayed in Table 3 for different dates before booting in the individual and pooled datasets. When applying the regression models to individual datasets, DATT_[850,730,675]_ and TI_NDRE[MEA]_ gave similar performances (DATT_[850,730,675]_: *R*^2^ = 0.57 to 0.69 for VE and *R*^2^ = 0.75 to 0.82 for SE, TI_NDRE[MEA]_: *R*^2^ = 0.56 to 0.67 for VE and *R*^2^ = 0.72 to 0.82 for SE), while NDTI_COR[850,730]_ performed poorly with *R*^2^ < 0.19 for VE and *R*^2^ < 0.67 for SE. After integrating the individual datasets, NDTI_COR[850,730]_ showed a close correlation with PNPA (*R*^2^ = 0.52 to 0.64), followed by DATT_[850,730,675]_ (*R*^2^ = 0.50 to 0.57), while TI_NDRE[MEA]_ yielded worse performance (*R*^2^ < 0.26). On the whole, excluding the VE data on March 24 with an *R*^2^ of only 0.57, SPSI generated the highest *R*^2^ values (VE: *R*^2^ = 0.61 to 0.74, SE: *R*^2^ = 0.76 to 0.87, pooled: *R*^2^ = 0.62 to 0.78) for any date in any dataset among all indices. Similarly, SPSI also exhibited the best validation performances with the highest *R*_V_^2^ and the lowest RMSE and RRMSE among all indices, except for the VE-specific model on March 24 (Table 4). Compared with the second best indices on each date, the *R*_V_^2^ values of SPSI increased by 0.11 to 0.23, and both RMSE and RRMSE decreased by 16.43% to 38.79% for the unified models applied to the pooled data.

**Table 3.** The regression equations and coefficient of determination (*R*^2^) of PNPA with 4 indices for VE data (*n* = 32), SE data (*n* = 54), and pooled data (*n* = 86) on different dates.

Date	Index	VE-specific model	*R* ^2^	SE-specific model	*R* ^2^	Unified model	*R* ^2^
2/23	DATT_[850,730,675]_	*y* = 1476.80*x* – 55.91	0.66^**^	—	—	—	—
	TI_NDRE[MEA]_	*y* = 2694.94*x* + 437.33	**0.67** ^**^	—	—	—	—
	NDTI_COR[850,730]_	*y* = 3261.77*x* + 376.02	0.19^*^	—	—	—	—
	SPSI	*y* = 1336.79*x* – 38.88	**0.67** ^**^	—	—	—	—
3/04	DATT_[850,730,675]_	*y* = 1359.18*x* – 63.27	0.57^**^	*y* = 2367.35*x* – 287.97	0.75^**^	*y* = 2012.72*x* – 214.45	0.50^**^
	TI_NDRE[MEA]_	*y* = 1749.46*x* + 322.15	0.56^**^	*y* = 3065.41*x* + 455.13	0.72^**^	*y* = 1797.76*x* + 436.44	0.26^**^
	NDTI_COR[850,730]_	*y* = 1042.36*x* + 408.16	0.07^ns^	*y* = 3619.47*x* + 378.48	0.57^**^	*y* = 3621.54*x* + 352.07	0.52^**^
	SPSI	*y* = 1248.68*x* – 70.29	**0.61** ^**^	*y* = 1609.07*x* – 99.39	**0.76** ^**^	*y* = 1596.36*x* – 138.97	**0.62** ^**^
3/14	DATT_[850,730,675]_	*y* = 1002.23*x* + 62.67	0.69^**^	*y* = 2087.34*x* – 238.23	0.75^**^	*y* = 1802.82*x* – 172.29	0.57^**^
	TI_NDRE[MEA]_	*y* = 1377.65*x* + 360.71	0.67^**^	*y* = 2711.53*x* + 601.32	0.76^**^	*y* = 780.30*x* + 516.54	0.10^**^
	NDTI_COR[850,730]_	*y* = 1157.18*x* + 390.94	0.17^*^	*y* = 2257.53*x* + 392.98	0.67^**^	*y* = 2351.96*x* + 363.60	0.64^**^
	SPSI	*y* = 893.76*x* + 57.15	**0.74** ^**^	*y* = 1311.04*x* – 42.27	**0.86** ^**^	*y* = 1318.66*x* – 77.52	**0.78** ^**^
3/24	DATT_[850,730,675]_	*y* = 885.14*x* + 72.21	**0.64** ^**^	*y* = 1629.97*x* – 83.29	0.82^**^	*y* = 1333.88*x* – 21.04	0.52^**^
	TI_NDRE[MEA]_	*y* = 1147.53*x* + 401.71	**0.64** ^**^	*y* = 2073.07*x* + 644.83	0.82^**^	*y* = 919.83*x* + 535.01	0.20^**^
	NDTI_COR[850,730]_	*y* = -900.40*x* + 453.96	0.04^ns^	*y* = 2373.38*x* + 428.52	0.51^**^	*y* = 2180.73*x* + 440.03	0.52^**^
	SPSI	*y* = 841.91*x* + 86.50	0.57^**^	*y* = 1207.63*x* + 11.67	**0.87** ^**^	*y* = 1233.43*x* – 30.92	**0.78** ^**^

Note: Significance level: no significance, ns; *P < 0.05; **P < 0.01. The highest *R*^2^ for each date in each column is highlighted in bold.

**Table 4.** *R*_V_^2^, RMSE (×10^4^/ha), and RRMSE (%) values for PNPA estimation from 4 indices (DATT_[850,730,675]_, TI_NDRE[MEA]_, NDTI_COR[850,730]_, and SPSI) with unified models and VE- or SE-specific models on different dates (VE: *n* = 16, SE: *n* = 27, unified: *n* = 43).

Date	Index	VE-specific model			SE-specific model			Unified model		
		*R*_V_^2^	RMSE	RRMSE	*R*_V_^2^	RMSE	RRMSE	*R*_V_^2^	RMSE	RRMSE
2/23	DATT_[850,730,675]_	0.60	42.86	9.68	—	—	—	—	—	—
	TI_NDRE[MEA]_	0.58	43.85	9.91	—	—	—	—	—	—
	NDTI_COR[850,730]_	0.19	60.98	13.78	—	—	—	—	—	—
	SPSI	**0.61**	**42.24**	**9.54**	—	—	—	—	—	—
3/04	DATT_[850,730,675]_	0.58	41.48	9.24	0.70	77.64	13.25	0.55	90.96	17.00
	TI_NDRE[MEA]_	0.59	40.91	9.11	0.67	81.19	13.86	0.34	110.39	20.64
	NDTI_COR[850,730]_	0.11	60.68	13.51	0.49	100.42	17.14	0.59	86.72	16.21
	SPSI	**0.61**	**40.29**	**8.97**	**0.70**	**76.56**	**13.07**	**0.71**	**72.47**	**13.55**
3/14	DATT_[850,730,675]_	0.67	40.57	9.20	0.79	64.36	10.99	0.70	75.98	14.28
	TI_NDRE[MEA]_	0.65	41.64	9.44	0.79	64.61	11.03	0.15	127.50	23.97
	NDTI_COR[850,730]_	0.28	59.64	13.52	0.60	89.62	15.30	0.64	83.47	15.69
	SPSI	**0.69**	**39.41**	**8.93**	**0.83**	**58.37**	**9.96**	**0.81**	**61.16**	**11.50**
3/24	DATT_[850,730,675]_	**0.65**	**35.94**	**8.03**	0.79	66.58	11.42	0.64	81.90	15.38
	TI_NDRE[MEA]_	0.63	37.38	8.35	0.76	70.34	12.07	0.28	116.58	21.89
	NDTI_COR[850,730]_	0	63.50	14.19	0.53	99.15	17.01	0.57	89.84	16.87
	SPSI	0.59	39.26	8.77	**0.89**	**47.90**	**8.22**	**0.87**	**50.13**	**9.41**

Note: The highest *R*_V_^2^ and the lowest RMSE and RRMSE for each model at each date are highlighted in bold.

The scatter plots of the measured versus predicted PNPA by all models of SPSI were distributed around the 1:1 line (Fig. S10). Nevertheless, some VE data points (inside the red ellipse) for the unified model were slightly overestimated (Fig. S10C) in comparison with that of the VE-specific model (Fig. S10A). Considering the estimation accuracy and the timing of field management measures, the optimal timing of PNPA estimation in winter wheat for any dataset was March 14 (the initial-jointing stage with reference to semi-winter wheat) by comparing all the models on different dates.

## Discussion

### PNPA estimation with SIs

Wheat AGB was linearly related to TN at individual stages, while PNPA was related to TN in a nonlinear (logarithmic) way over multiple stages (Fig. S1). Accordingly, the SIs for crop biomass estimation could be used to estimate PNPA. The performance of SIs varied with the wavelength configuration in PNPA estimation (Fig. 1). Specifically, the SIs based on the red-edge band outperformed those based on visible bands, which was consistent with the findings of Zheng et al. [16] and Cheng et al. [47]. The red-edge band was more sensitive to canopy structure and capable of detecting deeper canopy under high biomass conditions than the visible bands [48]. The 3-band SIs also exhibited better performances, which could be explained by the fact that the 3-band SIs obtaining more vegetation information have stronger saturation-resistant ability than the 2-band SIs [49]. However, the accuracy of the three-band vegetation index (TBVI_[850,730,450]_) was lower than that of NDRE due to the introduction of the blue band with stronger atmospheric effect [44]. In addition, the mathematical forms also affected the performance of SIs. For example, the optimized SAVI (OSAVI) performed worse than NDVI. The soil adjustment factor deteriorated the performance of OSAVI since all SIs were derived from green pixels.

PNPA could contribute the most to the final yield of wheat among all yield components [2]. Thus, the optimal date to estimate PNPA by SIs was April 8 (the initial-booting stage with reference to wheat planted in S2) for the SE (single-cultivar) dataset (Fig. S7B), which was in agreement with the optimal stage of wheat yield prediction determined by Ren et al. [50]. However, the optimal date to estimate PNPA by SIs was earlier for the VE (multi-cultivar) dataset (Fig. S7A), which could be caused by growth and development discrepancies among wheat cultivars with different vernalization responses and photoperiod sensitivities [51]. The springness cultivars required fewer days with the low temperature above 0 °C and the light duration to complete the vernalization and photoperiod phases; thus, their growth processes were faster than those of semi-winterness cultivars [51,52]. Additionally, the number of samples for springness cultivars (*n* = 30) was larger than that for semi-winterness cultivars (*n* = 18) in the multi-cultivar (VE) dataset. Moreover, the *R*^2^ values for various SIs increased and then decreased with the growth process (Fig. S7). This pattern coincided with the variation in vegetation signal represented by canopy spectral reflectance in the growing season [1]. The marginal differences in *R*^2^ from April 8 to May 9 (the booting stage to the initial-filling stage with reference to semi-winter wheat) could be related to the slight variations of SIs across the middle growth stages of crops under medium and high N rates [53]. This was the case for all samples in the VE dataset and two-thirds of samples in the SE dataset (Table 1 and Fig. S3). These results covering all critical growth stages not only demonstrated the optimal timing of PNPA estimated by SIs but also proved the feasibility of PNPA estimation before heading.

Relevant studies showed that many SIs tended to be saturated when monitoring crop AGB at high levels [16,18,19]. Additionally, there was a close AGB–TN–PNPA relationship (Fig. S1). Consequently, the spectral saturation also appeared in the estimation of wheat PNPA and affected the accuracies of the SI_OPT_ DATT_[850,730,675]_ (Fig. 2A and C). This phenomenon reveals the necessity of reducing spectral saturation to improve the estimation accuracy of wheat PNPA.

### Capacity of NDTI_COR[850,730]_ for reducing the spectral saturation

NDTI_COR[850,730]_ significantly reduced the saturation and exhibited a stronger correlation with PNPA than DATT_[850,730,675]_ in the subsets with severely saturated spectra (Fig. 4D to I). This result could be mainly caused by the green-pixel textural feature COR in NDTI_COR[850,730]_. Green-pixel COR exhibiting the strong saturation-resistant ability was different from the all-pixel textural features MEA, VAR, CON, and DIS examined in the biomass estimation of rice and wheat [16,29]. The textural features derived from green pixels described the difference in crop population structure more intensively than those from all pixels (Fig. S6). The complexity of the experimental dataset could also affect the performance of textural features. Specifically, the datasets used in relevant studies [16,29] were derived from only one type of interactive-factor experiment involving only 2 cultivars, while this research assembled 2 types of interactive-factor experiments involving 9 cultivars (Table 1). Although the 4 TIs derived from MEA, VAR, CON, and DIS performed well in the individual datasets, the best green-pixel TI_NDRE[MEA]_ yielded serious stratifications in the pooled dataset (Fig. S8 and Fig. 2D). Since COR is a measure of the linear dependency of pixel values among neighboring pixels in the grayscale image [21], the indices from green-pixel COR could distinguish different crop canopy structures and thus exhibited the saturation-resistant ability in estimating PNPA (Figs. 4G to I and 5). In addition, many studies have shown that the SIs composed of near-infrared and red-edge bands exhibited weaker saturation than those composed of near-infrared and visible bands in monitoring crop growth parameters (e.g., biomass and leaf area index) [17,47]. This also provided favorable evidence for the strong saturation-resistant ability of NDTI_COR[850,730]_ in PNPA estimation.

### Interpretation of the composite index SPSI

Recent studies have shown that the combination of spectral and textural information could improve the estimation accuracy of crop traits over the use of spectral or textural information alone [26,27,31]. Nevertheless, the way to combine spectral and textural information could affect the estimation of crop growth parameters [31]. This study proposed a composite index that is efficient and effective to combine the 2 types of information. Furthermore, uncovering the role of added textural information in the way of a TI in the composite index is also valuable for better understanding mechanism underlying the improvement. However, previous studies mostly used multiple regression or machine learning to combine the data and could not provide clear interpretations of the roles of added textural information [27,40]. In this study, DATT_[850,730,675]_ and NDTI_COR[850,730]_ were sensitive to the variation in PNPA at low values (< 400 × 10^4^/ha) and high values (> 400 × 10^4^/ha), respectively (Fig. 4J to K). Accordingly, the composite index SPSI constructed by the additive operation inherited the advantages of these 2 indices and reduced the spectral saturation in estimating PNPA (Fig. 4L).

In terms of the driving mechanism, the relationship between PNPA and SPSI was jointly affected by the AGB–DATT_[850,730,675]_ and PNPA–NDTI_COR[850,730]_ relationships (Fig. S11A and F to I), which can be seen from the process of SPSI construction. NDTI_COR[850,730]_ was determined directly based on the correlation of NDTI_COR_ with PNPA rather than AGB on the pooled data (Fig. 3). From an agronomic perspective, PNPA was directly related to TN and indirectly related to AGB (Fig. S1). In addition, DATT_[850,730,675]_ was well related to AGB, TN, and PNPA (*R*^2^ > 0.51; Fig. S11A to C). Therefore, the composite index SPSI was better related to PNPA than to TN and AGB (Fig. S11G to I).

Moreover, the choice of a mathematical operation is crucial for the performance of a composite index [28]. Although Liu et al. [29] constructed composite indices by fusing SIs and single-band textural features through multiplication or division, they also found that SI×MEA and SI/MEA indeed deteriorated the estimation of wheat biomass as compared to SIs. Consequently, the multiplicative and division operations are not suitable for integrating all types of textural features and SIs. Some studies have shown that the ratio-type and difference-type composite SIs had good performance in monitoring crop biochemical parameters (e.g., chlorophyll content) [44,54]. Nevertheless, the division and subtractive operations were used to reduce the sensitivity of the composite SIs to soil background or canopy structure and are inappropriate for combining the advantages of an SI and a TI. Zhang et al. [28] also found that multiplication and addition were the best mathematical operations to construct composite indices for combining the advantages of an SI and solar-induced chlorophyll fluorescence. Therefore, it is recommended to choose the appropriate mathematical operation for specific purposes while constructing the composite index.

### Advantages of SPSI in PNPA estimation

PNPA is closely related to crop growth status, which is seriously affected by cultivation factors [55,56]. The comprehensive effect of these factors caused the spectral saturation in the estimation of PNPA. The composite index SPSI performed well under 5 spectral uniformity scenarios (Fig. 5), which was attributed to the saturation-resistant ability of the textural component. Liu et al. [29] also demonstrated that the introduction of textural features made the composite indices delay the saturation point of leaf area index and show stronger relationships with wheat biomass as compared to SIs.

The canopy reflectance and canopy structure were different under various levels of cultivation factors [17,57]. This could explain why the performance of SPSI varied across cultivation factors at different levels (Fig. 6). Specifically, the SPSI had better performance than the spectral component in estimating PNPA only under high-level N fertilizer conditions among all N rates (Fig. 6A and E). This was probably because high-level biomass was often accompanied by high-level N fertilizer application [55], which was the dominant factor causing spectral saturation in crop biomass estimation among all N rates. In addition, the droop-leaf wheat often has relatively high canopy coverage since leaf direction seriously affects the canopy structure and light distribution [58]. In the same growth space, the droop-leaf wheat occupying more space in the horizontal direction is more prone to the spectral saturation problem than the erect-leaf wheat (Fig. S4). Therefore, the SPSI with the saturation-resistant ability produced higher accuracy than the spectral component for the droop-leaf type but not for the erect-leaf type (Fig. 6D and H).

Previous studies have shown that the combination of spectral and textural information could improve the estimation accuracy of crop traits [16,27]. This study also found that the complementarity of spectral and textural components enabled the composite index SPSI to perform best in estimating PNPA for any dataset among all indices (Tables 3 and 4). Even though NDTI_COR[850,730]_ performed poorly (*R*^2^ < 0.19) in the VE dataset, the composite index SPSI (*R*^2^ = 0.61 to 0.74) still performed better than DATT_[850,730,675]_ (*R*^2^ = 0.57 to 0.69) for 3 of the 4 dates (Table 3). The addition of NDTI_COR[850,730]_ also resulted in improvements in the SE dataset. In the pooled dataset, the improvement of SPSI over DATT_[850,730,675]_ in PNPA estimation was more significant. It was the complementarity of the spectral and textural components that enabled SPSI to perform best in any dataset among all indices.

### Limitations and prospects

Although SPSI has great potential in PNPA estimation under complex cultivation conditions, some limitations should still be taken into consideration. For example, it could be inferred from the construction process that the SPSI from April 8 would be not suitable for estimating PNPA. This could be attributed to the adverse effect of panicle emergence [59] and the changes in canopy structure resulting from the outcrop phenomenon of panicle for springness and early-sowed (S1) wheat in the experimental field on April 8. Additionally, the most sensitive textural feature to yield varied with the growth process, such as HOM for the booting stage and CON for the heading stage [40]. This research only used COR to construct SPSI and ignored the potential ability of other textural features in estimating PNPA. Therefore, future work should consider examining other textural features and extend the application of SPSI to the stages after heading (relatively uniform canopy structure).

In addition, the vegetation index thresholding approach was simple and effective for removing background materials from the UAV images with a 1.35-cm spatial resolution in this study. However, the changes in the threshold value and spatial resolution could affect the purity of extracted green pixels [60,61], which could bring uncertainty to the estimation of wheat PNPA. In particular, coarser spatial resolution and mixed pixels would make it more difficult to extract green pixels from satellite imagery. Linear spectral mixture analysis (LSMA) is an effective way to reduce the spectral mixing effect [62]. The abundance-adjusted vegetation index approach based on LSMA, which has been proven to be insensitive to the threshold value and spatial resolution, could reduce the modeling uncertainty [60]. Therefore, the abundance-adjusted SPSI may be constructed in future work for removing the background effect more efficiently in the UAV and satellite images with different spatial resolutions.

Furthermore, meteorological factors such as sunshine duration and temperature have important influences on the formation of wheat PNPA [63]. It is still unclear whether and how the use of meteorological factors could improve the accuracy and universality of PNPA estimation models. Recent studies have investigated the stacking of meteorological data, SIs, and textural features for improving the estimation of crop traits (e.g., N content and water content) [64,65]. The more efficient and interpretable integration of meteorological data and SPSI should be further explored in the future to develop robust models applicable to different sites and experimental conditions.

## Conclusions

This study proposed a new SPSI for reducing the spectral saturation and improving the accuracy of PNPA estimation in winter wheat before booting. Compared with the TIs derived from all pixels, the TIs derived from green pixels yielded better performance apart from TI_[HOM]_, TI_[ENT]_, and TI_[SEM]_ among all TI indices derived from 8 types of textural features. Except for SCE #2, SPSI significantly reduced the spectral saturation (*R*^2^ > 0.72, *P* < 0.05) in comparison with DATT_[850,730,675]_ (not significant) under 5 spectral uniformity scenarios. Compared with DATT_[850,730,675]_ (*R*^2^ = 0.61 to 0.76), SPSI exhibited lower sensitivities to cultivation factors and yielded better performances (*R*^2^ = 0.72 to 0.85) for the pooled data of each cultivation factor. Regardless of modeling and validation, SPSI produced the best performances for any date before booting in any data apart from the March 24 portion of the VE dataset among all indices. For the unified models applied to the pooled data, the *R*_V_^2^ values of SPSI increased by 0.11 to 0.23, and both RMSE and RRMSE decreased by 16.43% to 38.79% in comparison with the suboptimal index on each date. The optimal timing of PNPA estimation was March 14 (the initial-jointing stage with reference to semi-winter wheat) in multi-cultivar winter wheat. These findings demonstrated that it was effective to reduce the spectral saturation in PNPA estimation by introducing the textural information to the composite index. The new index SPSI could be beneficial for crop yield prediction with field phenotyping platforms. It also has great potential for improving PNPA estimation with high-resolution satellite imagery in the context of precision agriculture.

## Data Availability

The data that support the findings of this study are available from the corresponding author (T.C.) upon reasonable request.
