# Supplementary material for: SPSI: A Novel Composite Index for Estimating Panicle Number in Winter Wheat before Heading from UAV Multispectral Imagery
Source: Plant Phenomics. 2023 Sep 6;5:0087. doi: 10.34133/plantphenomics.0087 (PMC10482165; doi:10.34133/plantphenomics.0087)
Supplement: Supplementary 1 — Figs. S1 to S11 Tables S1 to S4 [file plantphenomics.0087.f1.docx]

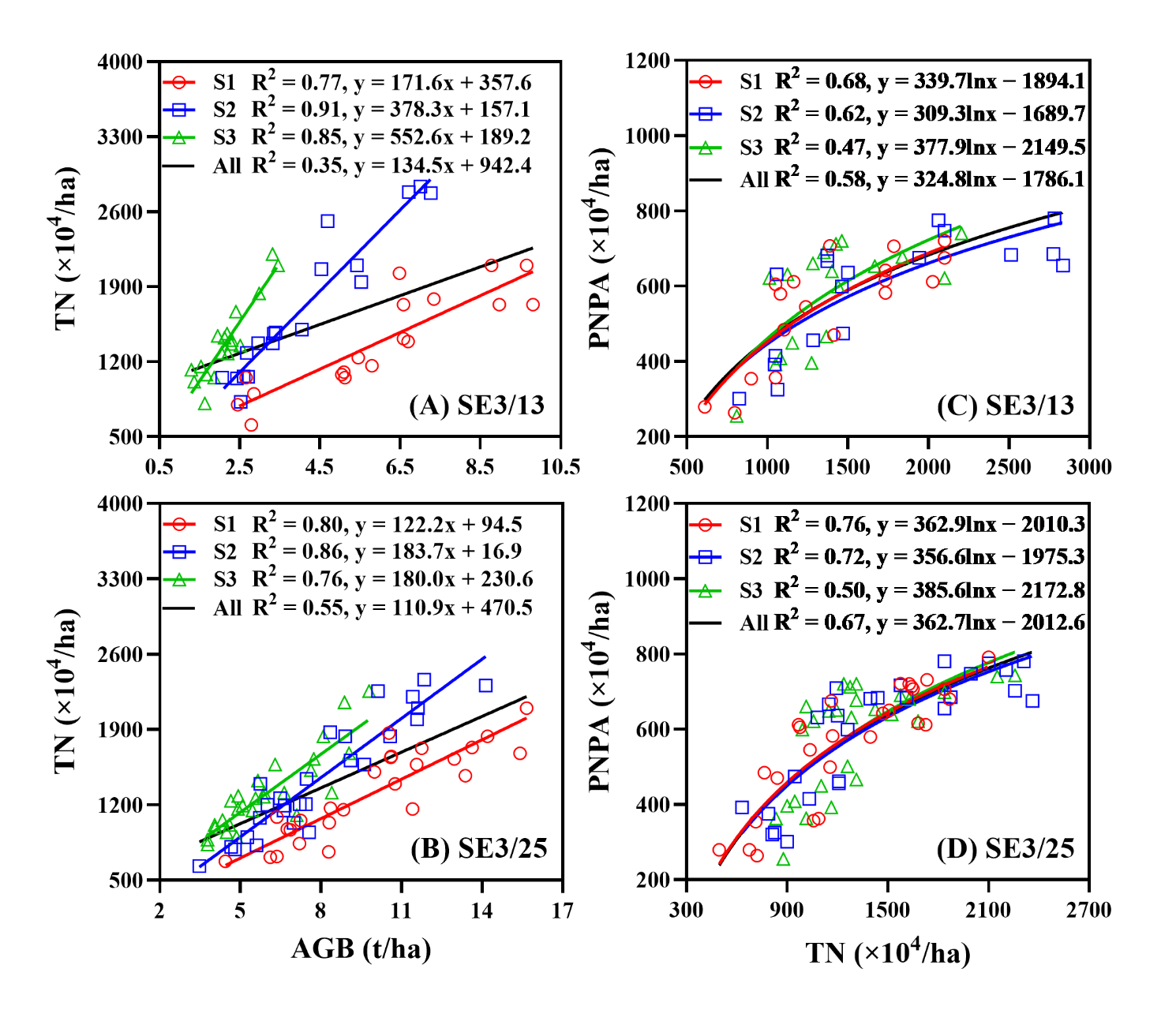


**Fig. S1**. The relationships of TN with AGB (A-B) and PNPA (C-D) in winter wheat under different sowing dates. Solid lines represent the fitted lines of all data points. Solid lines represent the fitted lines of all data points. TN: tiller number; AGB: aboveground biomass.


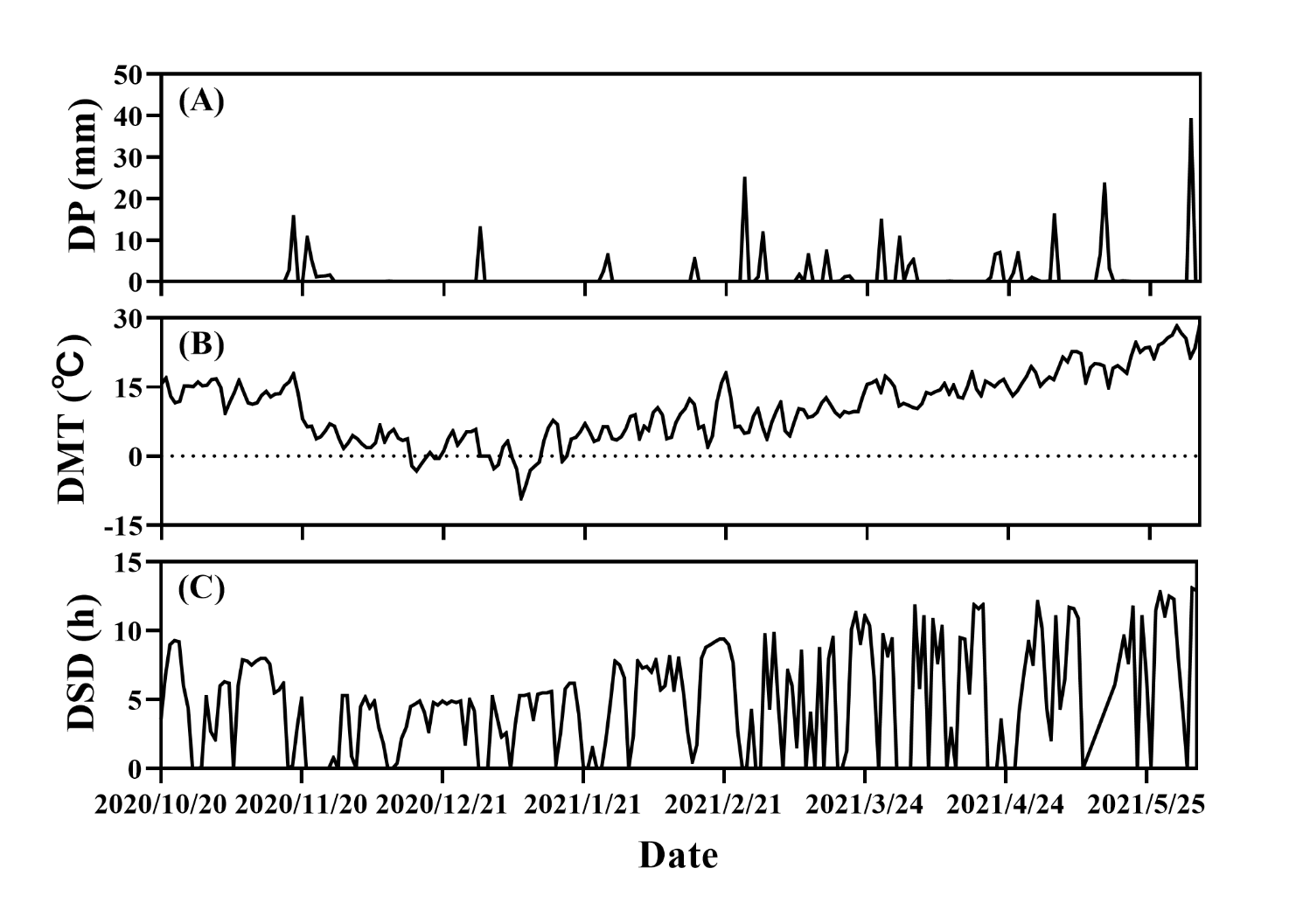


**Fig. S2**. The DP (A), DMT (B), and DSD (C) of the experimental site from 20 October 2020 to 5 June 2021. DP: Daily precipitation; DMT: Daily mean temperature; DSD: Daily sunshine duration.


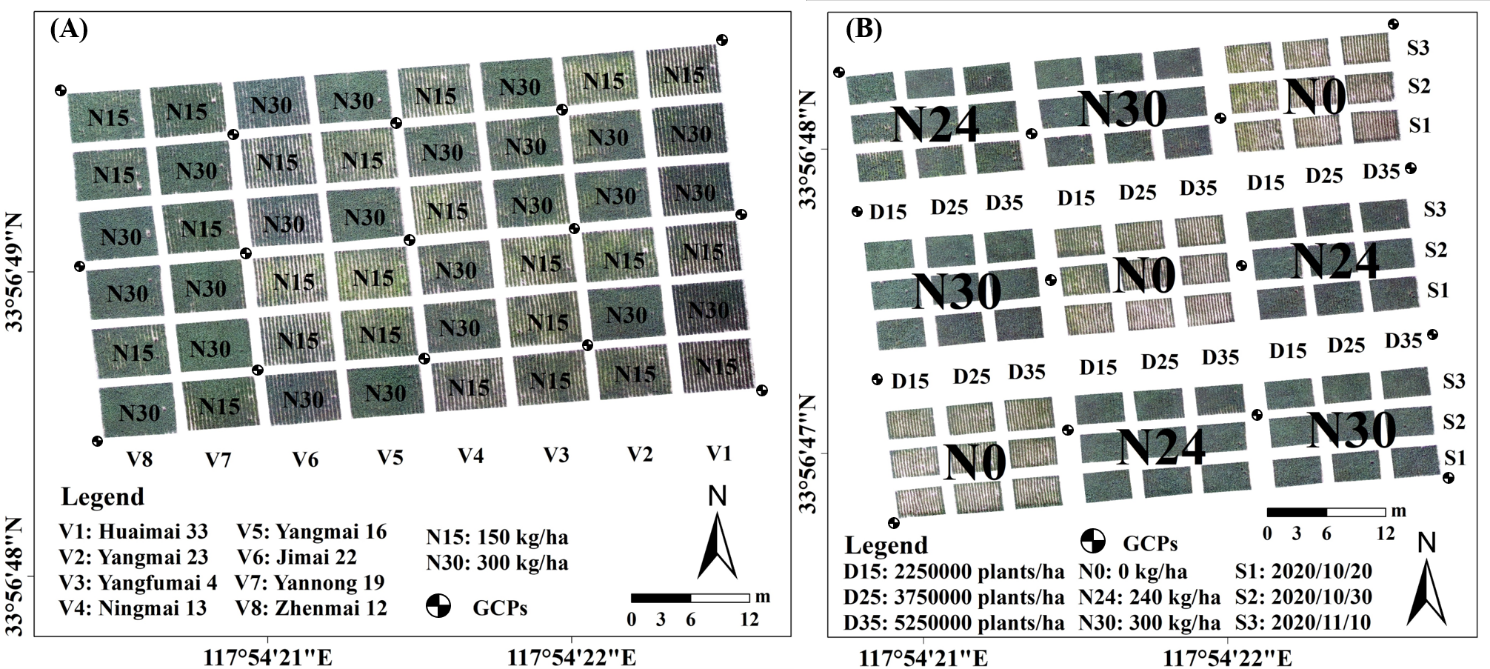


**Fig. S3**. The plot arrangements of the two experiments in 2020-2021. The orthophotos were captured with the UAV system on April 19, 2021. GCPs: Ground control points.


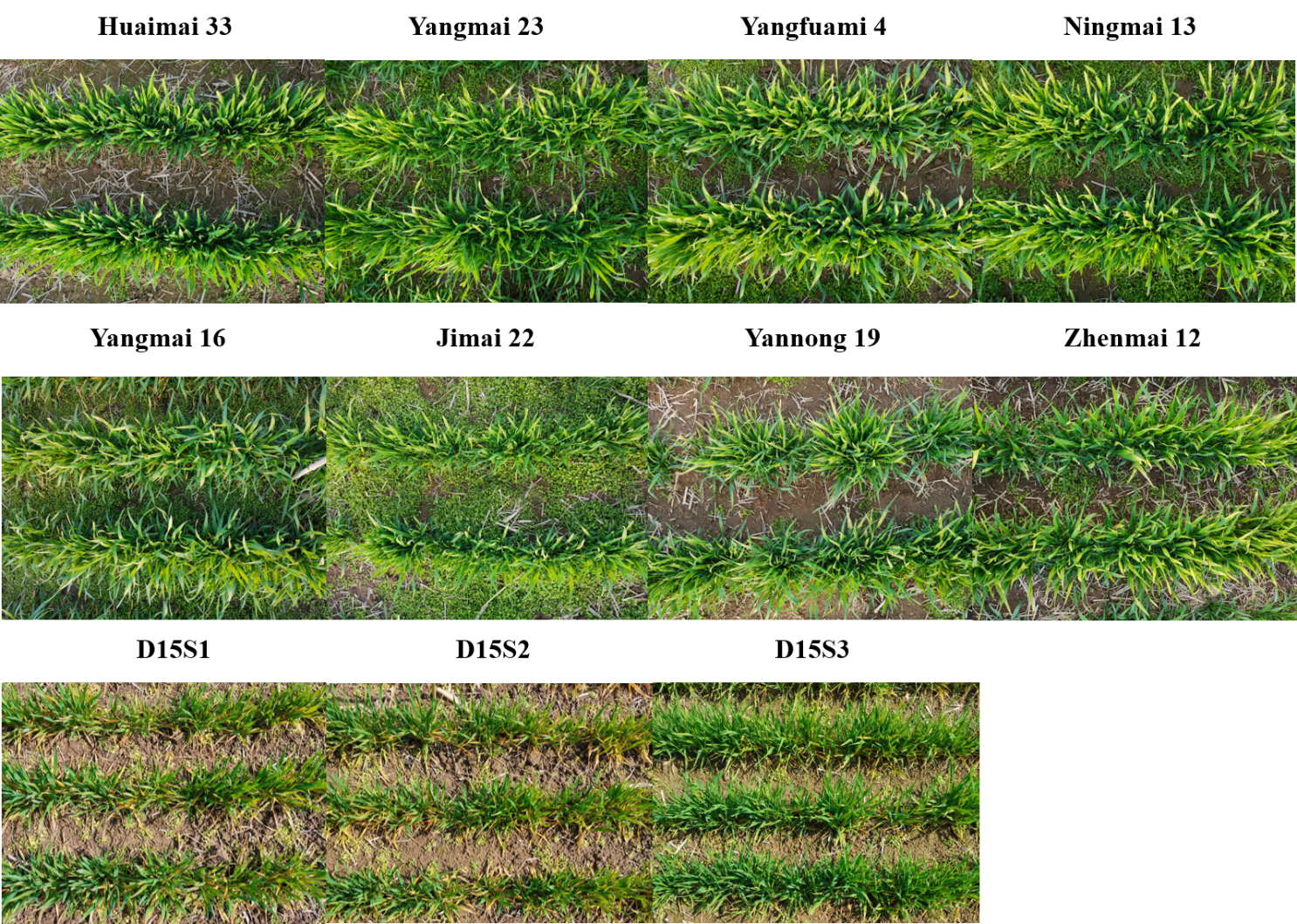


**Fig. S4**. Field photos for different wheat cultivars in Exp. 1 and for different sowing dates in Exp. 2 under N30 on March 2, 2021.


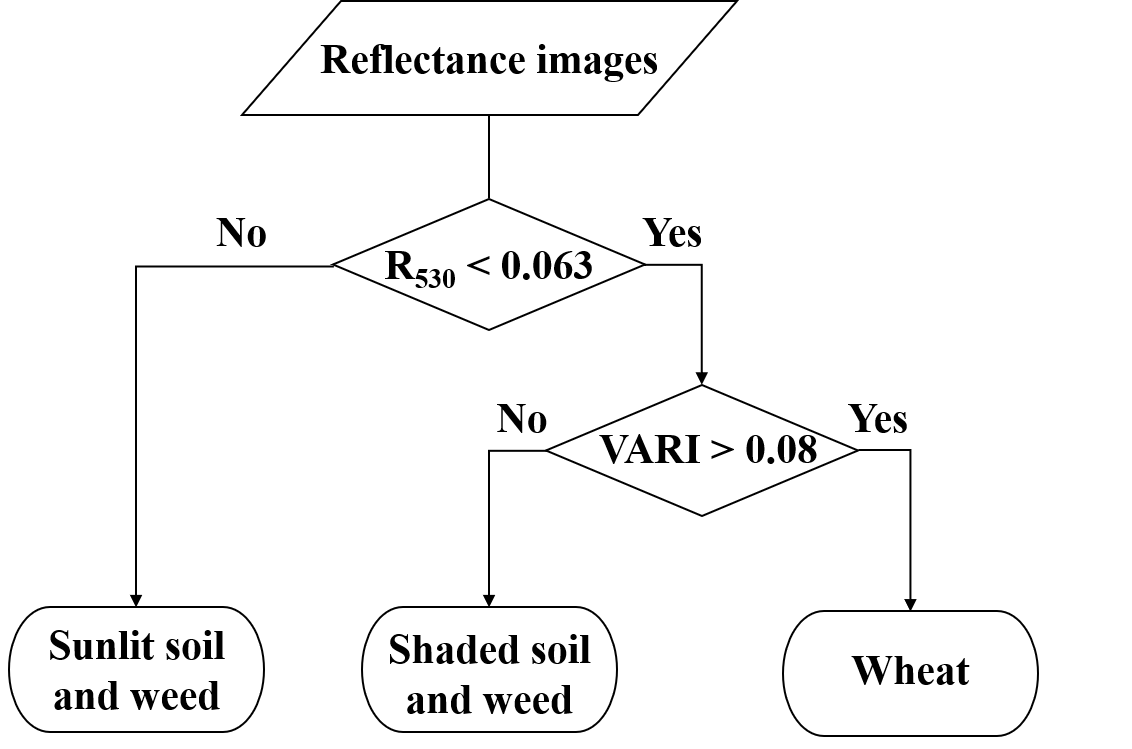


**Fig. S5**. Decision tree classification for extracting wheat pixels from the UAV imagery acquired on February 23, 2021.


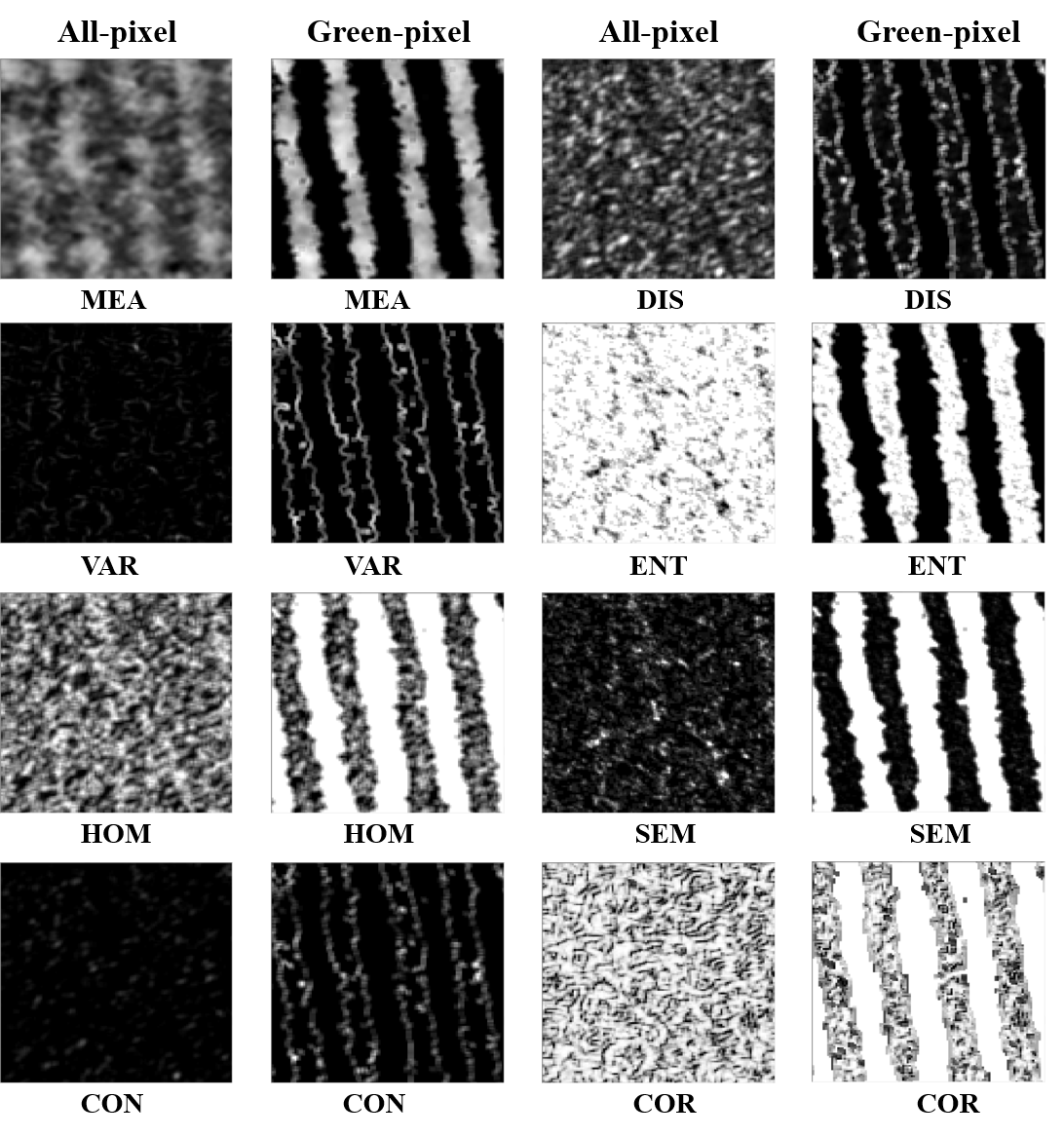


**Fig. S6**. Eight GLCM-based textural measurements of the 850 nm band derived from different pixel compositions (all-pixel or green-pixel) of the UAV multispectral imagery acquired on February 23, 2021.


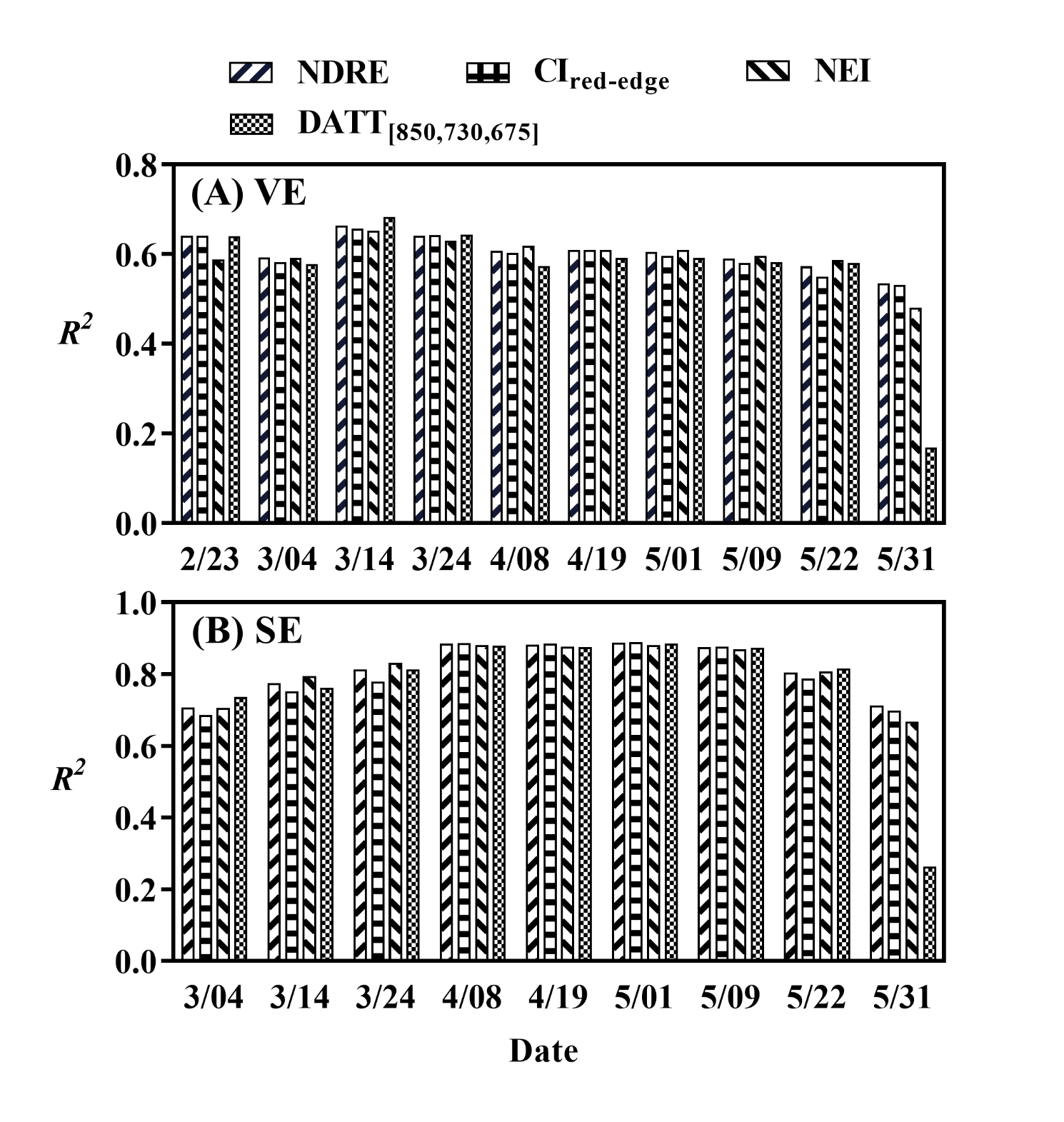


**Fig. S7**. *R^2^* values for linear relationships of PNPA with the four better SIs in winter wheat over individual dates for the (A) VE and (B) SE datasets.


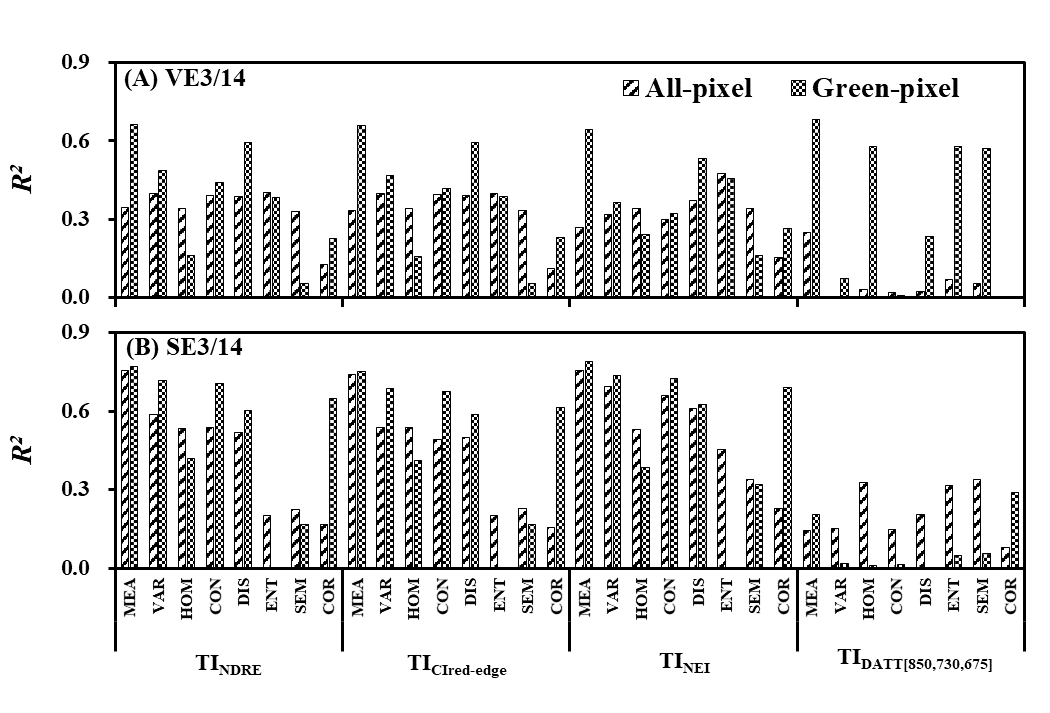


**Fig. S8**. *R^2^* values for linear relationships of PNPA with TIs based on the four better SI forms from different pixels for the March 14 portion of (A) VE and (B) SE datasets with the moderate coverage.


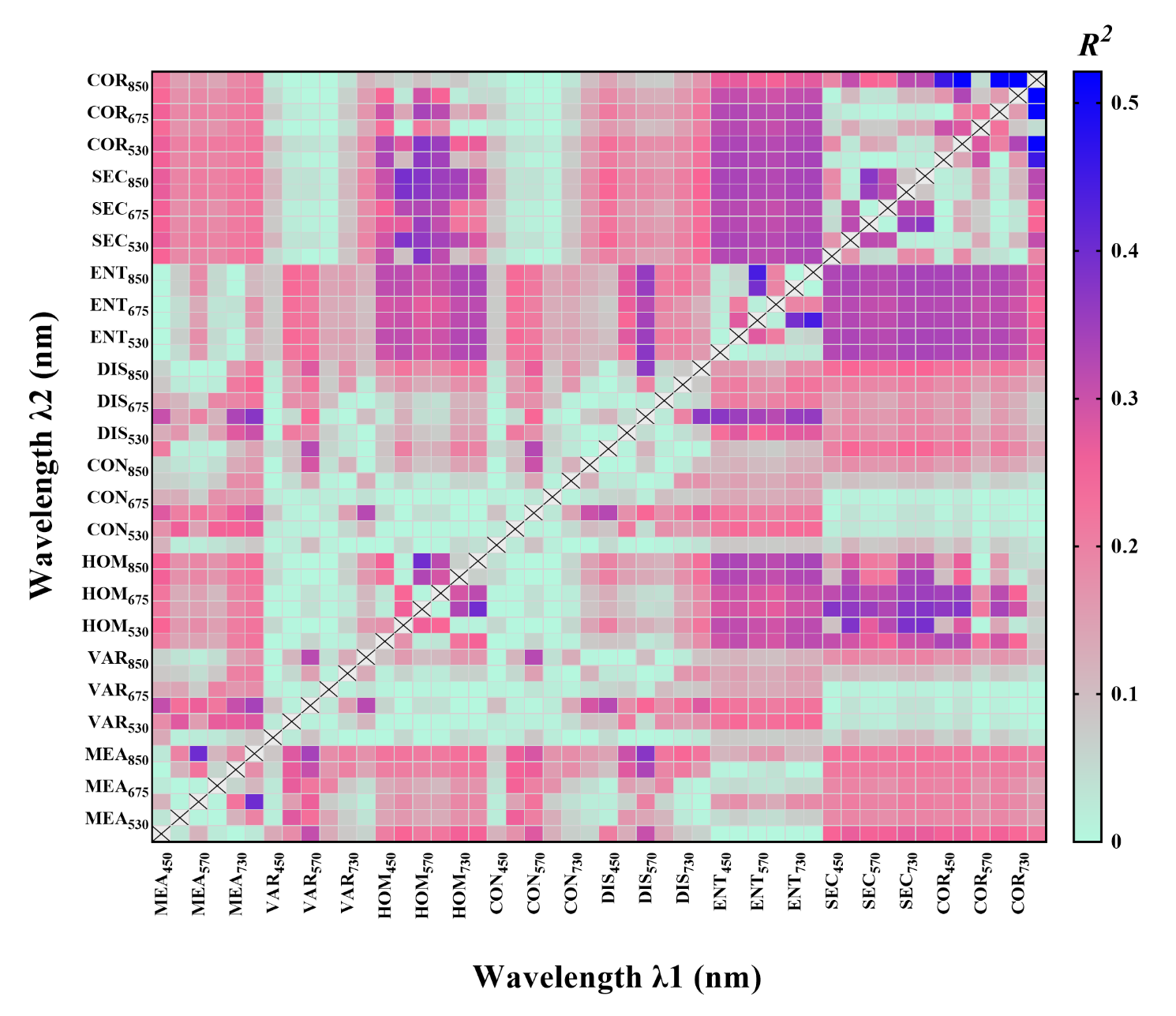


**Fig. S9**. *R^2^* for linear relationships between PNPA and the NDTI of all possible band combinations for the pre-booting portion of the pooled dataset. All textural features were derived from green-pixel textural images. The gray reticulated area represents invalid combinations.

**
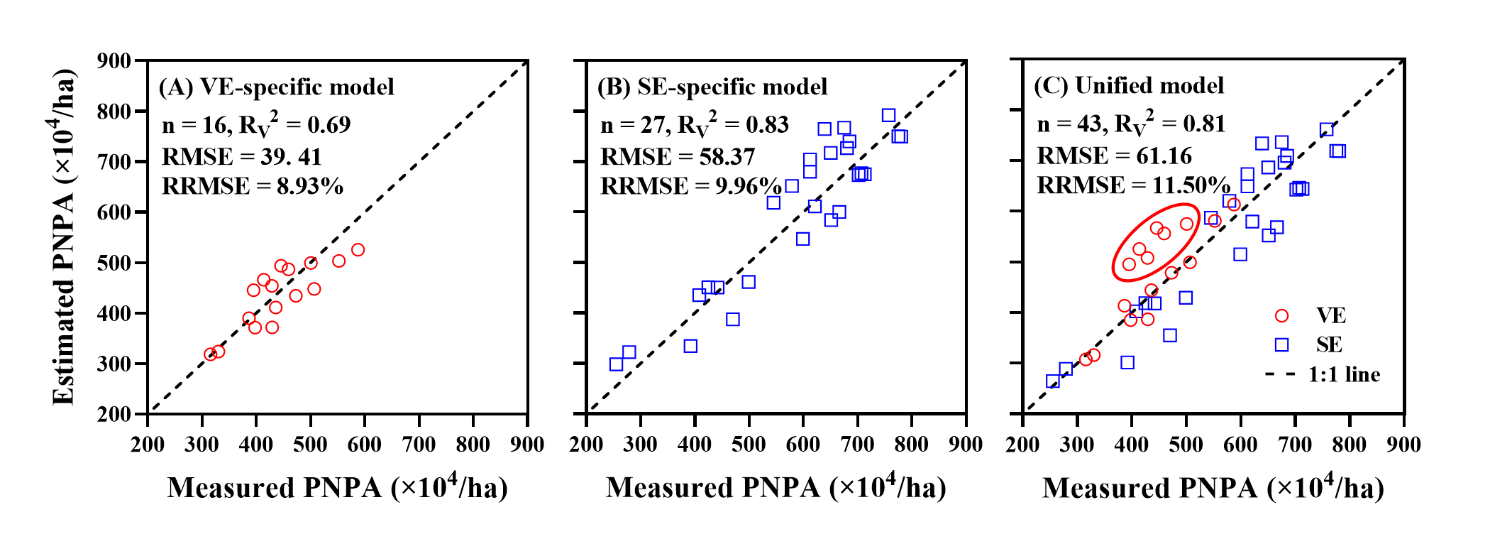
 Fig. S10**. Scatter plots of measured and estimated PNPA by SPSI with VE- (A) or SE-specific model (B) and unified model (C) on March 14.


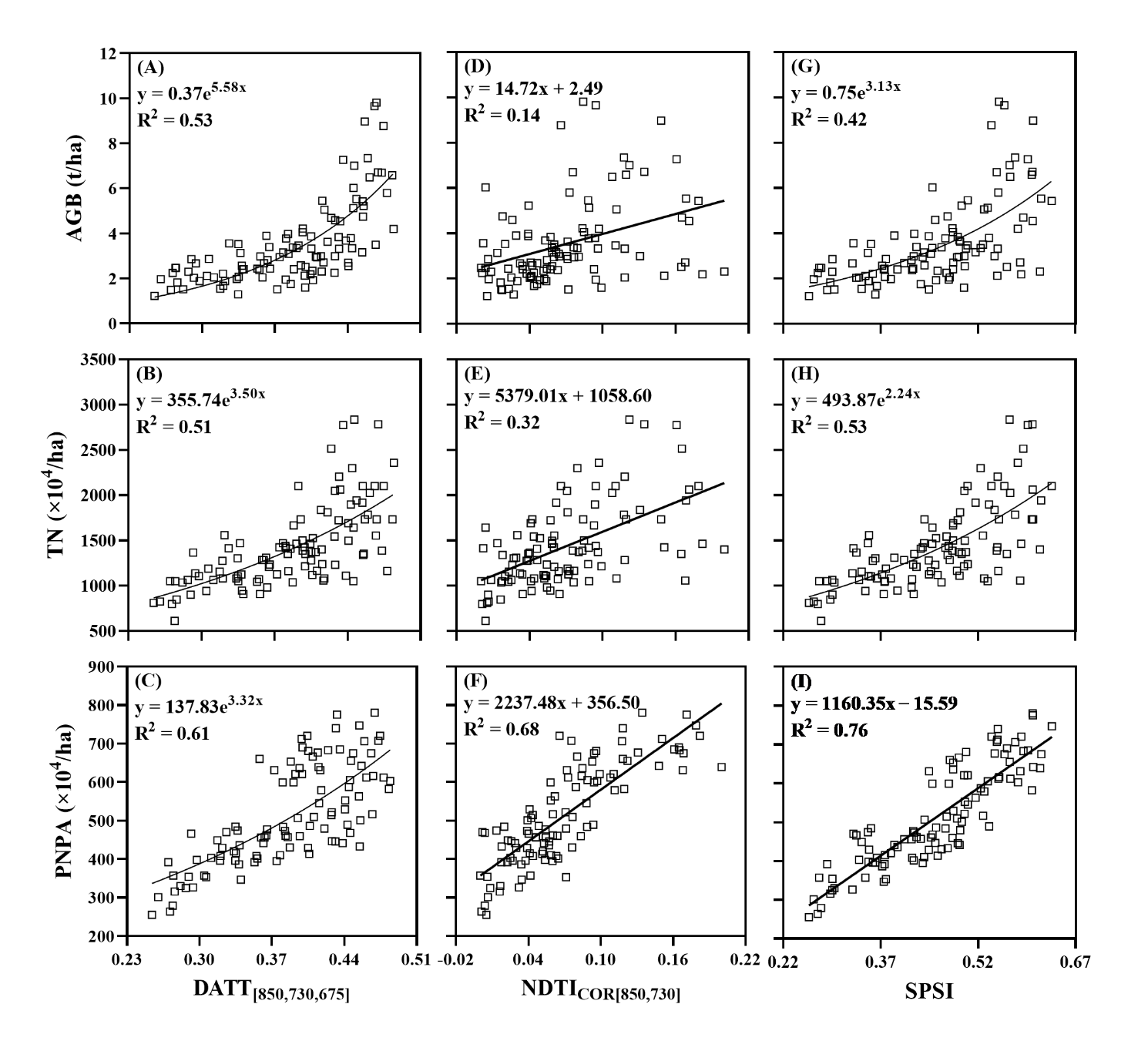


**Fig. S11**. The relationships of indices (DATT_[850,730,675]_ (A-C), NDTI_COR[850,730]_ (D-F), and SPSI (G-I)) with AGB (A, D, G), TN (B, E, H) and PNPA (C, F, I) in winter wheat for the samples from the March 14 portion of the pooled dataset. Solid lines represent the fitted lines of all data points. AGB: aboveground biomass; TN: tiller number.

**Table S1**. Dates of UAV flight for the two field plot experiments at critical growth stages.

| **Experiment** | **Date of UAV flight** | **Growth Stages** |
| --- | --- | --- |
| Exp. 1 | February 23, 2021^*^ | Regreening to standing |
| Exp. 2 | March 4, 2021 | Initial-standing to initial-jointing |
|  | March 14, 2021 | Late-standing to late-jointing |
|  | March 24, 2021 | Initial-jointing to initial-booting |
|  | April 8, 2021 | Late-jointing to late-booting |
|  | April 19, 2021 | Late-booting to late-heading |
|  | May 1, 2021 | Late-heading to initial-filling |
|  | May 9, 2021 | Late-anthesis to mid-filling |
|  | May 22, 2021 | Initial-filling to late-filling |
|  | May 31, 2021 | Late-filling to maturity |

**Note**: * denotes the field photos were acquired for only Exp. 1.

**Table S2**. Basic statistics on PNPA of winter wheat for the two field plot experiments.

|  |  | **PNPA (× 10^4^/ha)** | | | |
| --- | --- | --- | --- | --- | --- |
| **Exp. no.** | **Treatment** | **Mean** | **Median** | **RV** | **CV/%** |
| Exp. 1 |  |  |  |  |  |
|  | Cultivar |  |  |  |  |
|  | Huaimai 33 | 456.0^b^ | 462.5 | 395.0-516.4 | 10.4 |
|  | Yangmai 23 | 410.1^b^ | 424.6 | 326.4-472.9 | 14.5 |
|  | Yangfumai 4 | 390.5^b^ | 379.3 | 315.7-461.4 | 15.7 |
|  | Ningmai 13 | 448.3^b^ | 448.6 | 386.4-509.3 | 10.8 |
|  | Yangmai 16 | 408.7^b^ | 416.4 | 330.0-455.7 | 10.8 |
|  | Jimai 22 | 467.4^b^ | 471.8 | 395.7-529.3 | 10.7 |
|  | Yannong 19 | 556.8^a^ | 557.5 | 514.3-602.9 | 6.3 |
|  | Zhenmai 12 | 453.0^b^ | 443.9 | 428.6-500.7 | 6.0 |
|  | N rate |  |  |  |  |
|  | N15 | 412.3^b^ | 408.9 | 315.7-552.1 | 14.6 |
|  | N30 | 485.4^a^ | 478.2 | 401.4-602.9 | 10.1 |
| Exp. 2 |  |  |  |  |  |
|  | Sowing date |  |  |  |  |
|  | S1 | 564.3^a^ | 612.0 | 264.0-791.0 | 27.8 |
|  | S2 | 595.6^a^ | 666.0 | 301.0-780.0 | 26.5 |
|  | S3 | 581.3^a^ | 639.0 | 255.0-743.0 | 24.3 |
|  | Density |  |  |  |  |
|  | D1 | 529.1^b^ | 599.0 | 255.0-709.0 | 30.1 |
|  | D2 | 586.7^ab^ | 675.0 | 325.0-780.0 | 27.4 |
|  | D3 | 625.3^a^ | 655.0 | 449.0-791.0 | 18.9 |
|  | N rate |  |  |  |  |
|  | N0 | 387.4^b^ | 392.0 | 255.0-501.0 | 19.4 |
|  | N24 | 660.3^a^ | 655.0 | 545.0-747.0 | 7.8 |
|  | N30 | 693.4^a^ | 685.0 | 579.0-791.0 | 8.1 |

**Note**: RV: Range of variation; CV: Coefficient of variation. Values labeled by different lowercase letters represent the significant differences (*p* < 0.05) with the least significant difference (LSD) method.

**Table S3**. Number of pixels and running time of GLCM for different pixel components (all-pixel vs. green-pixel) of the UAV multispectral imagery acquired on March 14, 2021 for VE and SE.

| **Experiment** | **VE** | | **SE** | | |
| --- | --- | --- | --- | --- | --- |
| **Orthomosaic** | **All-pixel** | **Green-pixel** | **All-pixel** | **Green-pixel** | |
| Number of total pixels | 14,975,226 | 14,614,525 | 17,377,590 | 16,821,194 | |
| Number of pixels with PV = 0 | 2,527,020 | 9,303,021 | 2,882,220 | 12,952,345 | |
| Number of pixels with PV > 0 | 12,448,206 | 5,311,504 | 14,495,370 | 3,868,849 | |
| running time of GLCM (s) | 2,027 | 1,686 | 2,410 | | 1,807 |

**Note**: PV = Pixel value.

**Table S4**. Descriptions of the textural features.

| **Textural feature** | **Abbreviation** | **Meaning** |
| --- | --- | --- |
| Mean | MEA | The local mean value of greyscale quantization level in the processing window. |
| Variance | VAR | The local variance of greyscale quantization level in the processing window. |
| Homogeneity | HOM | The homogeneity of pixel values across an image. The value ranges from 0 to 1.0. |
| Contrast | CON | The local variation in pixel values among neighboring pixels. It is the opposite of HOM. |
| Dissimilarity | DIS | Similar to CON and inversely related to HOM. |
| Entropy | ENT | A measure of the randomness of pixel values. The value ranges from 0 to the Napierian logarithm of the processing window size. |
| Angular second-moment | SEM | A measure of the homogeneity for image grayscale distribution. The value ranges from 0 to 1.0. |
| Correlation | COR | Linear dependency of pixel values on those of neighboring pixels. The value ranges from -1.0 to 1.0. |
